# A negative feedback mechanism links *UBC* gene expression to ubiquitin levels by affecting RNA splicing rather than transcription

**DOI:** 10.1038/s41598-019-54973-7

**Published:** 2019-12-06

**Authors:** Marzia Bianchi, Rita Crinelli, Elisa Giacomini, Elisa Carloni, Lucia Radici, Emanuele-Salvatore Scarpa, Filippo Tasini, Mauro Magnani

**Affiliations:** Department of Biomolecular Sciences, University of Urbino Carlo Bo, 61029 Urbino, PU Italy

**Keywords:** Transcriptional regulatory elements, Ubiquitylation

## Abstract

*UBC* gene plays a critical role in maintaining ubiquitin (Ub) homeostasis. It is upregulated under stress conditions, and herein we report that it is downregulated upon Ub overexpression. Downregulation occurs in a dose-dependent manner, suggesting the existence of a fine-tuned Ub sensing mechanism. This “sensor” requires a conjugation competent ubiquitin to detect Ub levels. Searching the sensor among the transcription factors involved in basal and stress-induced *UBC* gene expression was unsuccessful. Neither HSF1 and HSF2, nor Sp1 and YY1 are affected by the increased Ub levels. Moreover, mutagenesis of their binding sites in the *UBC* promoter-driven reporter constructs does not impair the downmodulation effect. Epigenetic studies show that H2A and H2B ubiquitination within the *UBC* promoter region is unchanged upon ubiquitin overexpression. Noteworthy, quantification of nascent RNA molecules excludes that the downmodulation arises in the transcription initiation step, rather pointing towards a post-transcriptional mechanism. Indeed, a significantly higher fraction of unspliced *UBC* mRNA is detected in ubiquitin overexpressing cells, compared to empty vector transfected cells. Our findings suggest how increasing cellular ubiquitin levels may control the expression of *UBC* gene by negatively affecting the splicing of its pre-mRNA, providing a straightforward feedback strategy for the homeostatic control of ubiquitin pools.

## Introduction

The protein ubiquitin (Ub) is probably the most important post-translational modifier of the proteome in eukaryotic cells, regulating the stability, function, localization of its target substrates and as such, it controls an array of cellular processes and affects many signaling pathways^1,2^.

Ubiquitin has many peculiar features: (1) Ub is not encoded as a single polypeptide, but rather is translated as a fusion product either to ribosomal proteins or with multiple Ub moieties in tandem^3,4^; the precursor is processed by specific enzymes (DUBs) to give as final product Ub monomers^5^; (2) Ub is encoded by four different genes^6–8^ which ultimately yield the same product (monomeric Ub), but are not redundant in their functions, as demonstrated by the effects of selective knockout of the *UBB* or *UBC* locus^9–12^; (3) Ub exists inside the cell mainly partitioned into free and conjugated pools which are not static, but in dynamic equilibrium that changes to meet the changing cellular needs^13,14^; (4) Ub is one of the most abundant proteins, but surprisingly it is not produced in excess, as demonstrated by the upregulation of polyubiquitin coding genes *UBC* and *UBB*, under stressful conditions^9,15,16^. When the demand of Ub increases, e.g. during proteotoxic stress, besides the *de novo* synthesis of the protein and an improved Ub sparing from proteasomal degradation^17,18^, a redistribution of ubiquitin from histones to unfolded protein conjugates has been observed^19^. This “competition” between different Ub demanding processes reflects the limited pool of free Ub. This is also demonstrated by the evidence that, in yeast, Ub depletion may represent the main cause of toxicity induced by translational inhibitors^20^.

Given the involvement of Ub in many different cellular functions (in both normal and stressful conditions), maintaining Ub homeostasis is of paramount importance for every cell type and requires a highly dynamic but stringent regulation. In fact, it has been demonstrated that any alteration in Ub homeostasis, resulting in either an excess or a deficiency of free Ub, causes a “ubiquitin stress response”^21^. In particular, elevated Ub levels are intrinsic features of a variety of pathophysiological conditions, that upregulate Ub^22–25^, but may also derive from exogenous manipulation of cellular Ub levels, leading to ectopic Ub overexpression^9,20^. In a very recent paper, Han and coworkers^26^ developed a new system to increase the cellular Ub levels in a more physiological fashion; they used the CRISPR-Cas9 technology to induce upregulation of the endogenous *UBC* gene under normal conditions. The authors claim that this system may be useful to study the cellular response to an excess of Ub under normal conditions and to highlight if this prior upregulation of *UBC* may have a protective role towards incoming stress insults. Ubiquitin overexpression has been proved to be protective in the rescue from toxicity provoked by inhibitors of translation, which deplete free Ub by reducing its *de novo* synthesis^20^. On the other side, alteration of Ub homeostasis in mice, by overexpression of Ub in the neuronal compartment, impaired the synaptic function^27^. Moreover, when the authors investigated the potential effects of the higher Ub levels on the main components of the ubiquitin-proteasome system, they found a significant decrease in the expression of the endogenous polyubiquitin genes *UBC* and *UBB*, arguing that this is consistent with the need for a tightly regulated Ub homeostasis in neurons^25,27^. However, they did not further investigate the molecular mechanisms for this transcriptional downregulation. The need for a Ub sensor able to detect ubiquitin levels within the cell has been envisaged by different authors^9,18^, but the cellular component(s) able to fulfill this role have not been identified yet. In the present work we aimed to investigate the molecular mechanisms underlining *UBC* downregulation in Ub overexpressing cells. Indeed, we found that overexpression of wild-type ubiquitin in different human cell lines (both normal and tumor derived) resulted in lowered levels of *UBC* and *UBB* mRNAs; moreover, the *UBC* fold-decrease was directly related to the amount of ubiquitin overexpressed, suggesting that a proper negative feedback regulatory mechanism, able to sense the Ub levels, could act to maintain Ub within a defined concentration range under unstressed conditions. Another challenging issue is to highlight the *cis*-acting elements in the promoter region of *UBC* and *UBB*, which make these two genes the main targets and effectors of the ubiquitin sensing mechanism. The harvested data point towards a post-transcriptional ubiquitin-mediated modulation of *UBC* gene expression.

## Results

### Overexpression of ubiquitin downregulates the endogenous *UBC* gene expression

**Figure 1:**
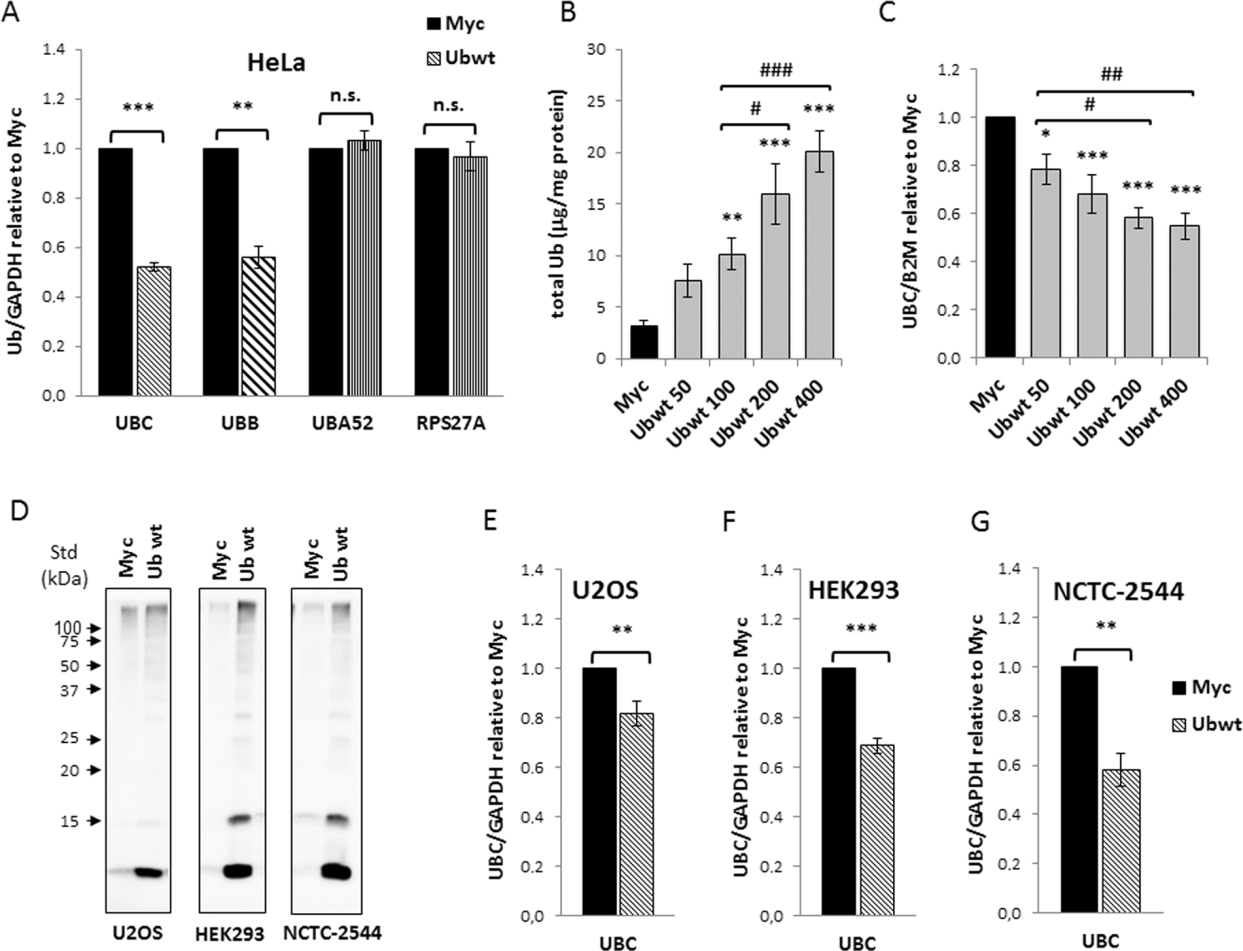
Overexpression of wild-type ubiquitin leads to *UBC* downregulation in different cell lines. (**A**) HeLa cells were transiently transfected with Ub wild-type expression vector (Ubwt) or the control empty vector pCMV-Myc (Myc). At 48 h post-transfection, the mRNA levels of the four Ub coding genes were determined by RT-qPCR (n = 12 each), normalized to *GAPDH*, and expressed as the fold change relative to the control Myc. (**B**) Quantification of total Ub content in whole cell lysates obtained from HeLa cells transfected with increasing amounts of Ubwt expression vector (n = 3). Total ubiquitin content was quantified after Usp2 digestion by solid phase immunoassay using purified bovine ubiquitin as standard. (**C**) Ub induces *UBC* downmodulation in a dose-dependent manner. RTqPCR (n = 6) of *UBC* mRNA in HeLa samples transfected as in (B); *UBC* was normalized to *B2M* and expressed as the fold change relative to the control Myc. (**D**) Western immunoblot analysis of U2OS, HEK293 and NCTC-2544 cells transfected with Ubwt or control vector pCMV-Myc, harvested 2 days post-transfection. Cell lysates, resolved by sodium dodecyl sulfate polyacrylamide gel electrophoresis (SDS-PAGE), were probed with anti-ubiquitin antibody. Arrowheads indicate molecular weight standards. (**E**,**F**,**G**) RTqPCR of *UBC* mRNA, respectively in U2OS (n = 14), HEK293 (n = 3) and NCTC-2544 (n = 3) cells harvested 48 h post-transfection with pCMV-Myc and Ubwt expression vectors. *UBC* mRNA levels were normalized to *GAPDH* and expressed as fold change versus the control Myc. Data are presented as means ± SEM from the indicated number of samples. ^*,#^p < 0.05; ^**,##^p < 0.01; ^***/###^p < 0.001 vs. control (Myc) or between two samples as indicated by horizontal bars. n.s., not significant.

Wild-type ubiquitin (Ubwt) was overexpressed in HeLa cells as a fusion product with a C-terminal Myc-tag, a strategy that reproduces the endogenous expression mechanisms^28^. Previous work has shown that Ub-transfected cells displayed a significantly higher Ub content (about 4-fold) compared to cells receiving the empty vector pCMV-Myc or left untreated, equally distributed between the free and conjugated pools^28^. To determine if ubiquitin overexpression had effects on its endogenous expression, we first examined the mRNA levels of the four Ub coding genes by RTqPCR. No significant changes in the *UBA52* and *RPS27A* transcripts were detected (Fig. 1A). In contrast, ubiquitin overexpression caused a significant decrease (around 50%) in the mRNA levels of the endogenous *UBC* and *UBB* genes (Fig. 1A). Transfection of different amounts of Ub construct resulted in an increase of total ubiquitin content which was strictly correlated to the quantity of transgene delivered^28^ (Fig. 1B). Downregulation of the *UBC* gene by exogenous Ub occurred in a dose dependent manner (Fig. 1C), starting from cells transfected with 50 ng of Ub plasmid, where the concentration of ubiquitin was ∼2.4-fold compared to the one detected in pCMV-Myc transfected cells, indicating that this regulatory loop may have a physiological relevance. To investigate whether *UBC* downregulation upon Ub overexpression was a general “buffer” mechanism to maintain Ub homeostasis, we transfected other cell lines with the Ubwt expression vector, namely NCTC-2544 and HEK293, which are normal cells and U2OS, which are tumor cells, but of different origin than HeLa. Immunoblotting analysis with anti-Ub specific antibody, at 48 h post-transfection, confirmed Ub overexpression in all treated samples (Fig. 1D). When the *UBC* gene transcript was detected by qPCR, an expression profile similar to HeLa was found for all the cell lines investigated (Fig. 1E–G).

### A conjugation competent ubiquitin is required for *UBC* downregulation

To explore how raising the intracellular ubiquitin levels caused a significant decrease of *UBC* mRNA, we hypothesized the presence of a cellular “ubiquitin sensor” capable of detecting the ubiquitin pool dynamics and affecting the expression of two out of the four Ub coding genes. Ubiquitin signals in many different ways: substrate modifications range from a single ubiquitin molecule to complex polymeric chains, with different types of ubiquitylation often eliciting distinct outcomes^29^. While Lys48-linked polyUb acts notoriously as proteasomal degradation signal, Lys63-linked Ub chains have various non-proteolytic roles^30,31^. To test whether *UBC* downregulation relies on a ubiquitin and proteasomal dependent degradation event or on a non-proteolytic Ub-mediated mechanism, HeLa cells were transfected with two ubiquitin mutants containing single lysine to arginine mutations, respectively at position 48 (UbK48R) and 63 (UbK63R), in parallel with the Ubwt expression plasmid and the empty vector pCMV-Myc. To further address if ubiquitin conjugation to the target “sensor(s)” was required to elicit *UBC* downmodulation or if, alternatively, non-covalent Ub binding was involved, we developed three new ubiquitin mutants: UbG76A, carrying an alanine residue instead of glycine at position 76 (this mutant has been reported to interfere with the activity of deubiquitylating enzymes)^32,33^; UbΔGG, a mutant ubiquitin lacking the two C-terminal glycine residues (which cannot be conjugated to other proteins but can be ubiquitinated to generate unanchored ubiquitin chains in the cell)^34^; UbI44A, carrying a mutation in the hydrophobic patch (L8, I44 and V70) that is critical for Ub interaction with many partner proteins and the proteasome^35^.

**Figure 2:**
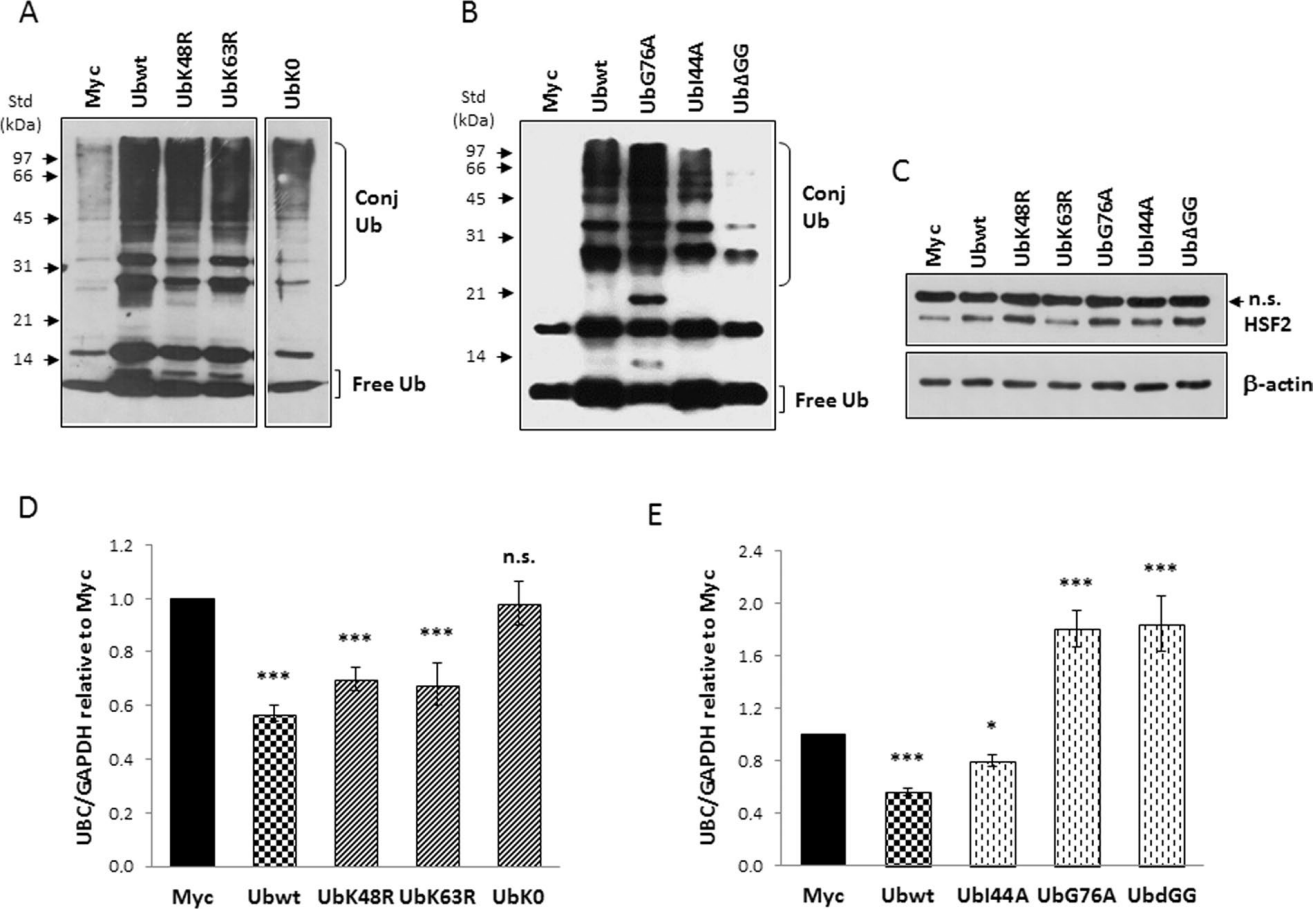
A conjugation competent ubiquitin is required for *UBC* downregulation. (**A**,**B**) Western blot analysis of HeLa cells transiently transfected with wild-type Ub or the different Ub mutants, in parallel with the control empty vector pCMV-Myc, harvested 48 h post-transfection. Cell lysates, resolved by SDS-PAGE, were probed with anti-ubiquitin antibody. Arrowheads indicate the molecular weight standards. (**C**) HSF2 levels in whole extracts obtained from HeLa cells transfected with the indicated constructs. Actin was detected as loading control and is shown in the lower panel. (**D**,**E**) *UBC* mRNA levels were determined, in HeLa cells transfected with the indicated constructs, by RTqPCR (n = 6 each), normalized to *GAPDH* and expressed as the fold change versus the control Myc. Data presented in (**D**,**E**) are means ± SEM from the indicated number of samples. *p < 0.05; ***p < 0.001 vs. control (Myc); n.s., not significant. The images shown in (**A**,**B**,**C**) are representative of three independent experiments. Vertical spaces inserted between lanes in panel A indicate removal of intervening, irrelevant samples. Full-length immunoblots are presented in Supplementary Fig. S5.

All ubiquitin mutants were expressed at high levels, as demonstrated by the increase in the immunoreactive signal compared to that detected in Myc-transfected cells (Fig. 2A,B). Moreover, some differences in the Ub distribution between the free and conjugated pools were appreciable consistently with the expected mechanism of action of the analogues (Fig. 2A,B). Specificity of interference with the ubiquitin pathway was demonstrated by the accumulation of HSF2 protein in cells overexpressing the Ub analogues compared to cells transfected with the Ubwt construct or the empty vector pCMV-Myc, with the only exception represented by UbK63R transfected cells (Fig. 2C). This is in agreement with several reported observations that K63-linked polyubiquitination of proteins does not affect their degradation kinetics^36,37^. Of note, no significant differences in the HSF2 content were found between Ubwt and Myc transfected cells (1.05 ± 0.05 versus Myc set as 1; n = 3) (Supplementary Fig. S1).

RTqPCR analysis of *UBC* mRNA revealed a statistically significant repression of gene transcription by UbK48R and UbK63R (p < 0.001 versus Myc), indicating that the K48- and K63-assembled Ub chains were not involved (Fig. 2D). In contrast, the downregulation effect was lost in cells transfected with UbK0, a lysine-less ubiquitin mutant, theoretically incapable of forming any polyubiquitin chain (Fig. 2D). On the whole these results suggest that a Ub poly-chain based on lysines other than K48 and K63 may be involved. Transfection of UbI44A resulted in *UBC* downregulation, as occurred with wild-type Ub (Fig. 2E). By contrast in cells overexpressing UbG76A or UbΔGG we found, instead of downregulation, an induction (about 1.8-fold versus Myc) of *UBC* expression (Fig. 2E). It is known that UbG76A is conjugated less efficiently than wild-type Ub and once incorporated cannot be deconjugated^32,33^. A strong accumulation of Ub-conjugated proteins was indeed detected in UbG76A expressing cells compared to Ubwt and UbI44A transfected cells (Fig. 2B). Conversely, UbΔGG cannot be conjugated^34^, but it can be the substrate for the building of free polyUb chains which are evident in the blot of Fig. 2B as immunoreactive bands with an electrophoretic mobility consistent with the molecular weight of di- (about 17 kDa), tri- (about 26 kDa) and tetra-Ub (about 34 kDa) polymers. Although these two mutants have different mechanisms of interference with the ubiquitination machinery, they share the ability to sequester the endogenous wild-type Ub into the conjugated fraction, thus they are expected to cause depletion of the free Ub pool which could in turn be responsible for *UBC* induction (Fig. 2E). Unfortunately, the depletion effect cannot be appreciated from the blot by comparing the ubiquitin pattern of UbG76A and UbΔGG expressing cells with Myc-transfected cells, since the mutants have been deliberately expressed as untagged proteins to avoid tag interference, thus they are indistinguishable from endogenous Ub.

### Promoter analysis by transfection of reporter constructs reveals the importance of the *UBC* intron for the downmodulation effect

**Figure 3:**
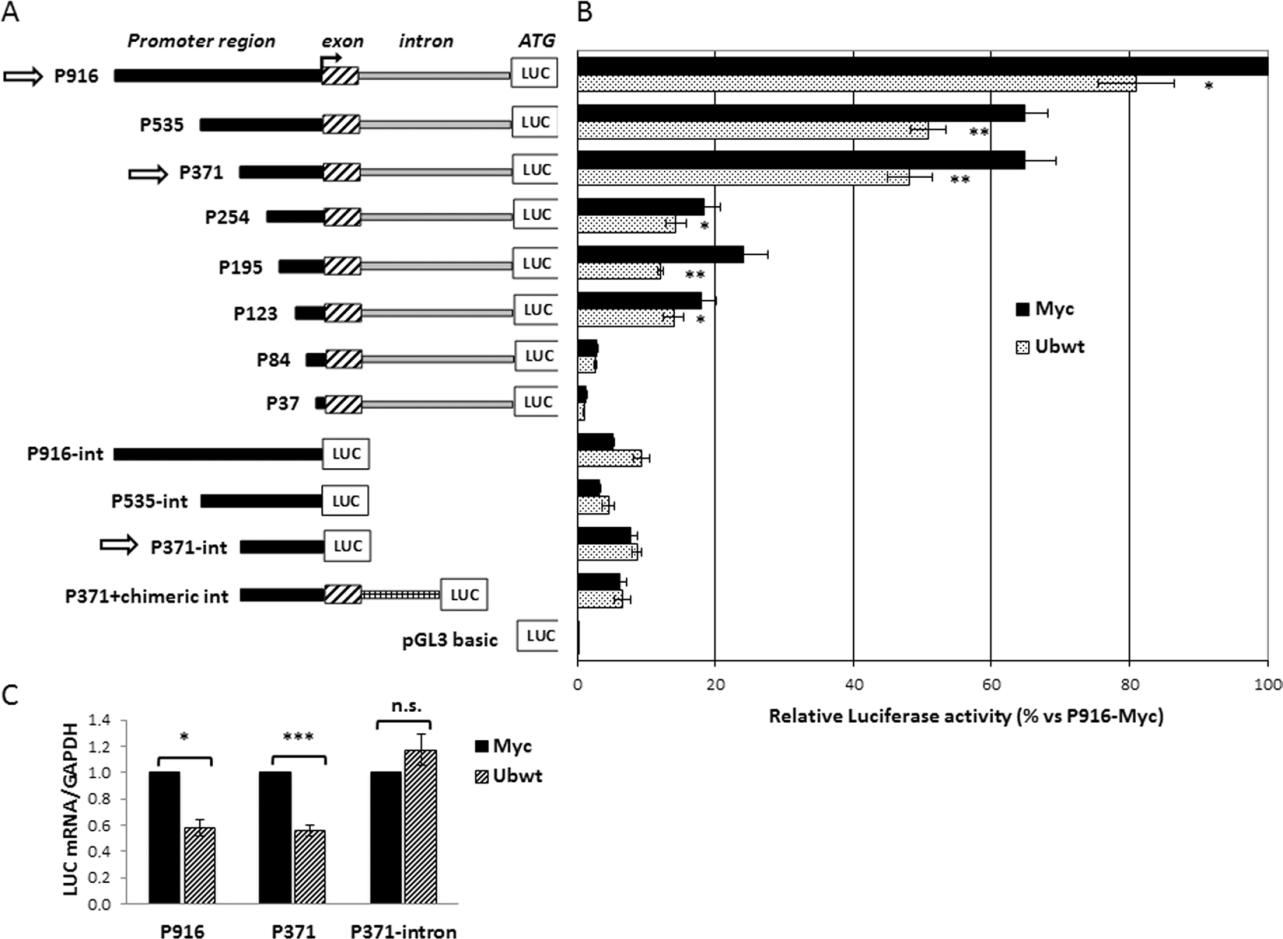
Effect of ubiquitin transfection on *UBC* promoter-driven luciferase expression. (**A**) Diagram of the 5′- and 3′-nested deletions of *UBC* promoter constructs. At the top there is the longer construct (P916) bearing 916 bp of the promoter region upstream of the TSS, the first non coding exon and the unique *UBC* intron, before the luciferase coding sequence (LUC). The name of the reporter vectors indicates the length of the upstream promoter region included (from −916 to −37). The contructs “–int” are devoid of the intron sequence, while in the P371+ chimeric int construct, a heterologous chimeric intron replaces the endogenous *UBC* intron. pGL3-basic is the promoter-less luciferase vector, used as control. (**B**) HeLa cells were transiently cotransfected with the reporter constructs shown in (A) and the Ubwt expression vector or the empty vector pCMV-Myc. Forty-eight hours post-transfection cells were harvested and luciferase activity was determined and normalized against total protein concentration. All values were referred to the sample cotransfected with P916/pCMV-Myc, set arbitrarily to 100 (n = 3). (**C**) Luciferase mRNA expression of the intron-bearing (P916 and P371) and intron-less (P371-int) constructs after overexpression of ubiquitin in HeLa cells. LUC mRNA was detected by RTqPCR and normalized to *GAPDH*. The value obtained in Ub overexpressing cells was expressed relative to the Myc transfected sample, set to 1 (n = 8). Data presented in (B,C) are means ± SEM from the indicated number of experiments. *p < 0.05; **p < 0.01; ***p < 0.001 vs. control (Myc); n.s., not significant.

To narrow down the promoter region responsible for the *UBC* gene responsiveness to the increase in cellular ubiquitin levels, we cotransfected different 5′ and 3′ serially deleted promoter constructs, with the Ubwt expression vector or the pCMV-Myc empty vector. Most of the luciferase reporter constructs have been previously described^38,39^ while others (P254, P195, P123 and P84) were developed during this study (Fig. 3A). They differ for the length of the upstream promoter region (5′ deletions) and the presence or not of the *UBC* intron (3′ deletions). The luciferase activity was given for each reporter vector with respect to the value obtained for the largest construct cotransfected with the empty vector (P916 + Myc), set as 100%. This preliminary screening highlighted that promoter downmodulation was detectable for all the intron bearing constructs, with the exception of the constructs with longer 5′ deletions (P84 and P37) which exhibited a very low promoter activity (Fig. 3B). It is remarkable that intron elimination not only drastically weakened the *UBC* promoter activity, but also resulted in the lack of ubiquitin-driven downregulation. Significantly, replacement of the endogenous *UBC* intron with a chimeric intron (P371 + chimeric int), PCR-amplified from the phRL-CMV vector (Promega) as previously described^38^, did not restore the downregulation effect upon Ub overexpression. The promoter activities of P916 and P371 vectors, which possess similar activity under basal conditions^38^, and of the intron-less version of P371 were determined, after Ub overexpression, by measuring the luciferase mRNA levels, besides luciferase enzymatic activity. In fact, due to its robustness and high sensitivity, RTqPCR is certainly a more appropriate and direct method to measure promoter-driven transcription and also allows to exclude any possible interference of ubiquitin with post-transcriptional mechanisms controlling luciferase protein levels^40,41^. As shown in Fig. 3C, luciferase mRNA expression of constructs P916 and P371 lowered when ubiquitin levels raised, while there was no significant change in the intron-less construct driven luciferase expression, suggesting that the downregulation event requires the presence of the intron.

Previous studies by our team^38,39,42^ and others^43^ have identified the *cis*-*trans* elements which mediate *UBC* promoter activity under basal and stressful conditions. Briefly, YY1 binding sites have been found in the intron and the upstream promoter sequence^39^. Likewise, Sp1 binding motifs are positioned both upstream of the transcription start site (TSS)^43^ and within the intron sequence^38,39^, while three Heat Shock Elements (HSEs) were characterized in the proximal and distal promoter region^16,42^. Both YY1 and Sp1 transcription factors (TFs) were found to sustain basal *UBC* gene expression, while HSF1 and HSF2 mediate *UBC* induction upon different stresses. These findings, which represent the starting background for the present study, are summarized in the schematic diagram of Supplementary Fig. S2A.

### Role of HSF1 and HSF2 in *UBC* downregulation

**Figure 4:**
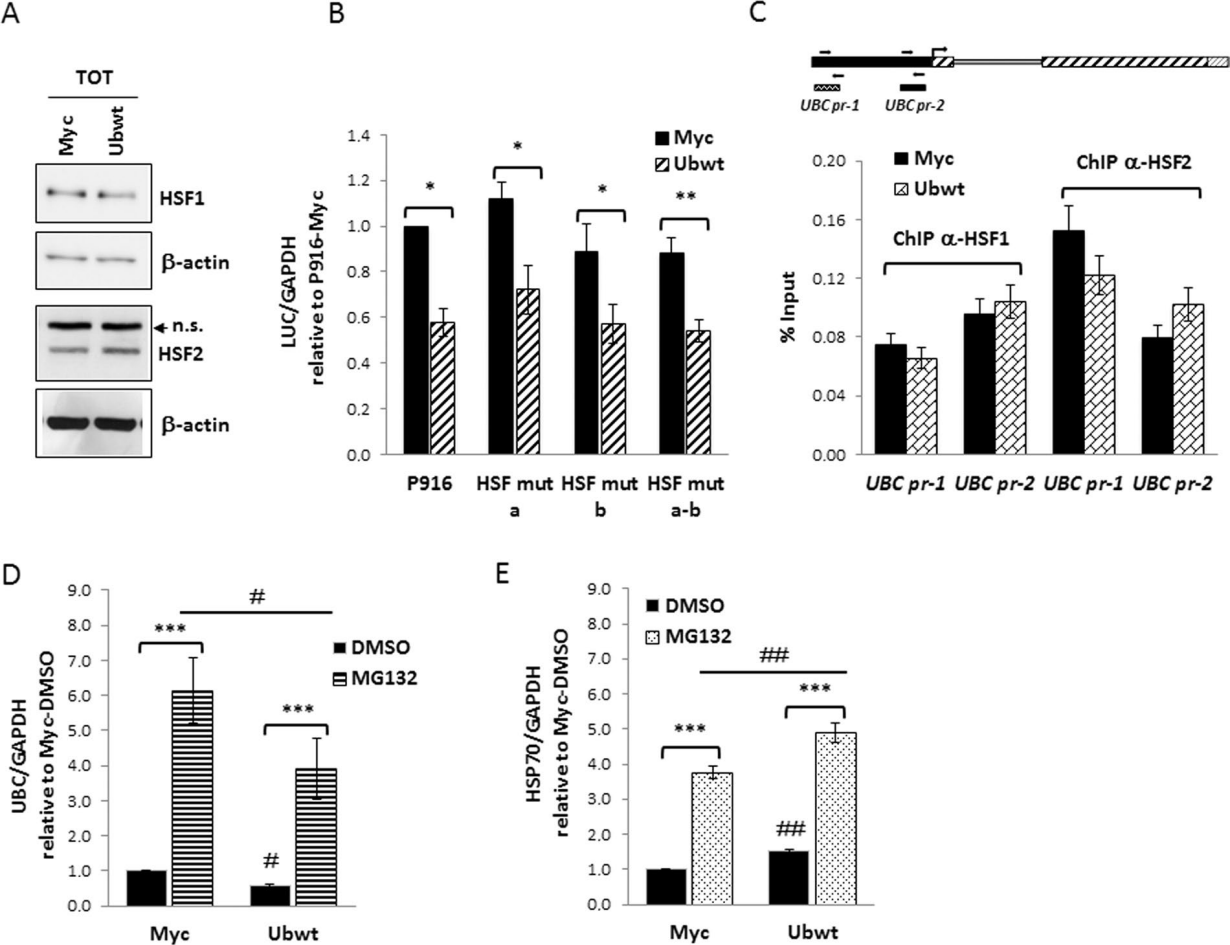
Investigating the role of HSF1 and HSF2 transcription factors in *UBC* downregulation. (**A**) Immunoblot analysis of HSF1 and HSF2 protein factors in whole extracts obtained from HeLa cells transiently transfected with the Ub expression vector (Ubwt) or the empty control vector (Myc). β-actin was used as loading control (lower panels). (**B**) The wild-type reporter construct P916 and the HSF mutant counterparts (HSF mut a, b, a-b) were cotransfected in HeLa cells with the Ub expression construct or the empty vector pCMV-Myc. Forty-eight hours post-transfection, luciferase mRNA levels were determined by RTqPCR, normalized to *GAPDH* and expressed as fold change versus the P916/Myc cotransfected sample, set to 1 (n = 4). (**C**) ChIP analysis of Myc and Ubwt transfected cells, using antibodies specific for HSF1 and HSF2; for the internal IP control, no Ab was added. RealTime PCR was carried out on chromatin before (Input) and after immunoprecipitation (ChIPed DNA) using selected primer pairs which amplify the *UBC* promoter regions (outlined above the graph) containing the previously identified HSEs (*UBC* pr-1 and *UBC* pr-2, respectively). Data are expressed as % of chromatin input controls, calculated using the formula 2^−ΔCt^ × 100. Results shown in the histogram represent the mean ± SEM of two independent experiments, assayed in duplicate. (**D**,**E**) Myc and Ubwt transfected cells were treated with 20 μM MG132 or the vehicle DMSO as control for 4 h. *UBC* (**D**) and *HSP70* (**E**) mRNA levels were determined by RTqPCR (n = 4), normalized to *GAPDH* and expressed as the fold change relative to the control (Myc/DMSO). Data presented in (**B**-**E**) are means ± SEM from the indicated number of experiments. ^*,#^p < 0.05; ^**,##^p < 0.01; ^***^p < 0.001 vs. control (Myc) or between two samples as indicated by horizontal bars. n.s., not significant. The images shown in (**A**) are representative of three independent experiments. Full-length immunoblots are presented in Supplementary Fig. S6.

Both HSF1 and HSF2 undergo ubiquitin and proteasome dependent degradation^44–46^. In light of the above, we wondered if the downregulatory effect on *UBC* gene activity, in ubiquitin overexpressing cells, could be mediated by these two members of the Heat Shock Factor family. We measured HSF1 and HSF2 total levels in pCMV-Myc and ubiquitin transfected cells by western immunoblotting and found no difference (Fig. 4A). To further investigate the possible role of these HSFs in the *UBC* downregulation, we took advantage of available reporter vectors where luciferase expression is driven by a *UBC* promoter sequence with intact HSEs (P916 wt) or with these sites mutagenized, one at a time or in combination^42^ (see Supplementary Fig. S2B). Cotransfection of these reporter constructs with the empty or Ub expression vector revealed that mutagenesis of HSF binding sites did not affect the downmodulation effect (Fig. 4B). These data were also confirmed by ChIP experiments showing that HSF1 and HSF2 *in vivo* occupancy of the *UBC* promoter did not change upon ubiquitin overexpression (Fig. 4C).

Moreover, having demonstrated that HSF1 and HSF2 drive *UBC* induction under proteotoxic stress triggered by transient proteasome inhibition^42^, we challenged ubiquitin overexpressing cells with MG132 to check what happened to *UBC* expression. As shown in Fig. 4D, *UBC* transcription was induced upon MG132 treatment both in Myc and Ubwt transfected cells, with a similar fold change versus the corresponding DMSO treatment, used as control. The same behavior was found for *HSP70* mRNA, detected as representative HSF1 target gene (Fig. 4E). On the whole, these results show that cells respond to MG132 treatment by inducing *UBC*, despite the high intracellular Ub levels, meaning that the Heat Shock Response (HSR) and the downregulation effect, both impacting on *UBC* gene expression, are distinct (not intersecting) regulatory mechanisms. HSF1 and HSF2 are not the Ub sensor we were looking for.

### Role of Sp1 in *UBC* downregulation

**Figure 5:**
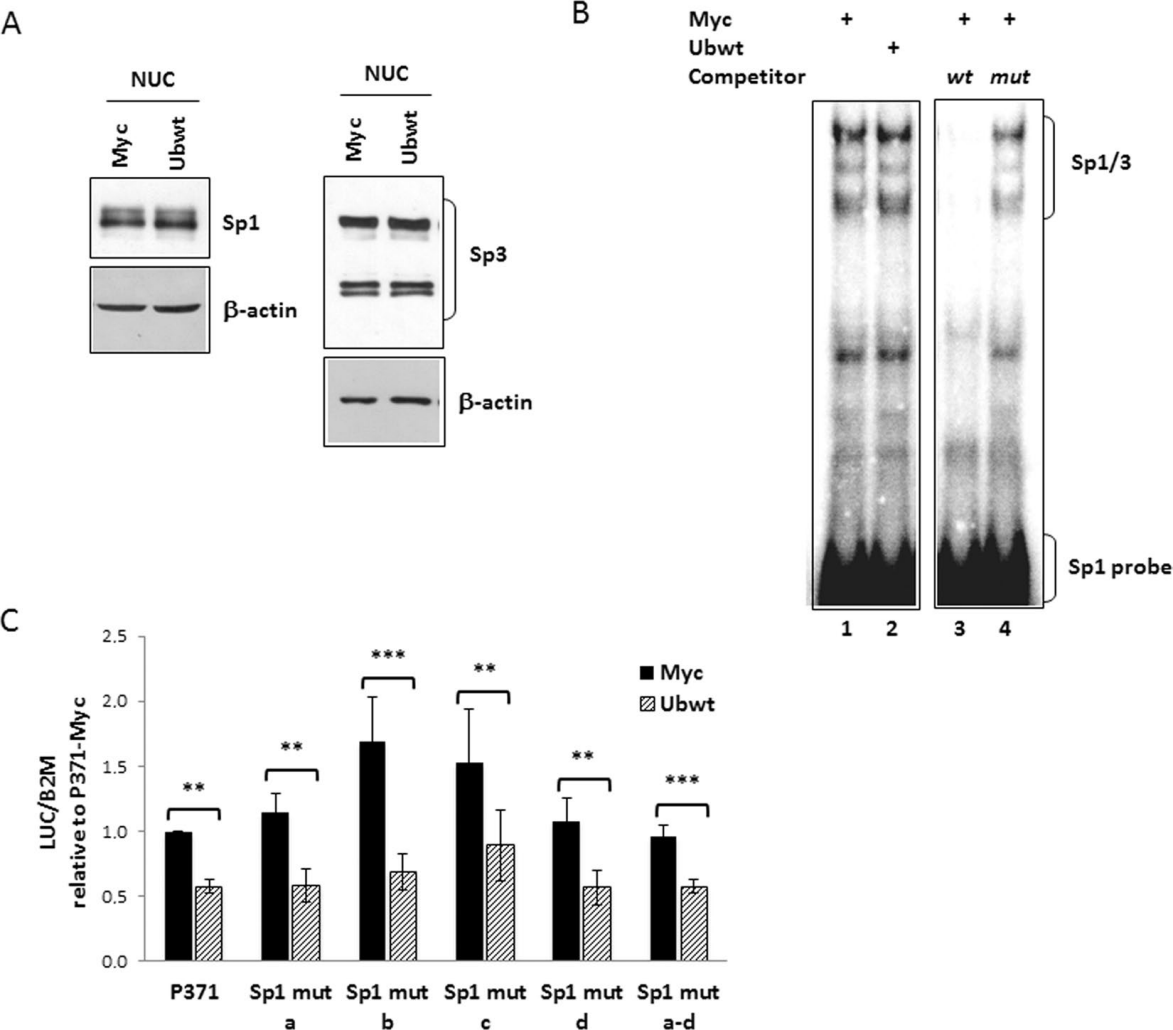
Investigating the role of Sp1 and Sp3 transcription factors in *UBC* downregulation. (**A**) Immunoblot analysis of Sp1 and Sp3 protein factors in nuclear extracts obtained from HeLa cells transiently transfected with the Ub expression vector (Ubwt) or the empty control vector (Myc). β-actin was used as loading control. (**B**) EMSA using, as probe, a ^32^P-labeled oligonucleotide containing the tandem binding sites for Sp1 identified in the −319/−280 region of *UBC* promoter^43^. Protein/DNA complexes were detected upon incubation of probe with nuclear extracts (5 μg) from cells transfected as in (**A**) (lanes 1–2). Specificity of retarded bands was assessed by competition experiments where nuclear extracts from Myc-transfected cells were preincubated with a 50-fold excess of cold wild-type or mutant h*UBC* Sp1 competitor (lanes 3–4). (**C**) The wild-type reporter construct P371 and the Sp1 mutant counterparts (Sp1 mut a, b, c, d, a-d) were cotransfected in HeLa cells with the Ub expression construct or the empty vector Myc. Forty-eight hours post-transfection, luciferase mRNA levels were determined, normalized to *B2M* and expressed as fold change versus the P371/Myc cotransfected sample, set to 1 (n = 4). Data presented in (**C**) are means ± SEM from the indicated number of experiments. **p < 0.01; ***p < 0.001 vs. control (Myc). The images shown in (**A**,**B**) are representative of three independent experiments. Vertical spaces inserted between lanes in panel B indicate removal of intervening, irrelevant samples. Full-length immunoblots and EMSA are presented in Supplementary Fig. S7.

To explore the potential role of Sp1 in the *UBC* downregulation, we determined the Sp1 levels in the nuclear fractions of pCMV-Myc and Ubwt transfected cells by Western blot analysis and found no significant difference (Fig. 5A). The same occurred for Sp3 (Fig. 5A), another member of the Sp family of transcription factors, with similar DNA binding properties as Sp1, as supported by our evidences that Sp3 occupies, at least *in vitro*, the same binding sites as Sp1 in the *UBC* promoter^38^. To assess if the higher cellular ubiquitin levels affected the ability of Sp1 to bind to its target sites in the *UBC* promoter, rather than modulating its protein levels, we performed a bandshift assay using an oligonucleotide containing the tandem binding sites for Sp1 identified in the *UBC* promoter (−319/−280 region)^43^. Incubation of the ^32^P-labeled DNA duplex with nuclear extracts derived from both pCMV-Myc and Ubwt transfected cells resulted in the appearance of several protein/DNA complexes, with a typical Sp1/Sp3 pattern^39^ (Fig. 5B). Protein binding specificity was assessed by competition experiments where nuclear extracts from Myc transfected cells were pre-incubated with an excess of unlabeled ODN containing the wild-type Sp1 consensus sequence which was able to prevent protein complex formation while a mutagenized competitor sequence was not (Fig. 5B). But most importantly, no difference in the pattern and/or intensity of retarded bands could be appreciated between ubiquitin-overexpressing and control cells. These results are in agreement with those obtained by cotransfection of luciferase reporter constructs with the Ub expression vector: both P371 and P254 reporter vectors (carrying and lacking, respectively, the upstream Sp1 binding sites) were downregulated by ubiquitin overexpression (Fig. 3B). Concerning the Sp1 binding sites previously identified within the intron sequence^39^, when either the single site Sp1 mutants (Sp1 mut a, b, c, d) or the reporter vector with mutations in all Sp1 binding motifs (Sp1 mut a-d), were cotransfected with the Ub expression plasmid, they exhibited a reduction of luciferase mRNA expression similar to the wild-type construct P371 (Fig. 5C). Taken together, the evidences obtained indicate Sp1 as not involved in the ubiquitin-driven downmodulation of *UBC*.

### Role of YY1 in *UBC* downregulation

**Figure 6:**
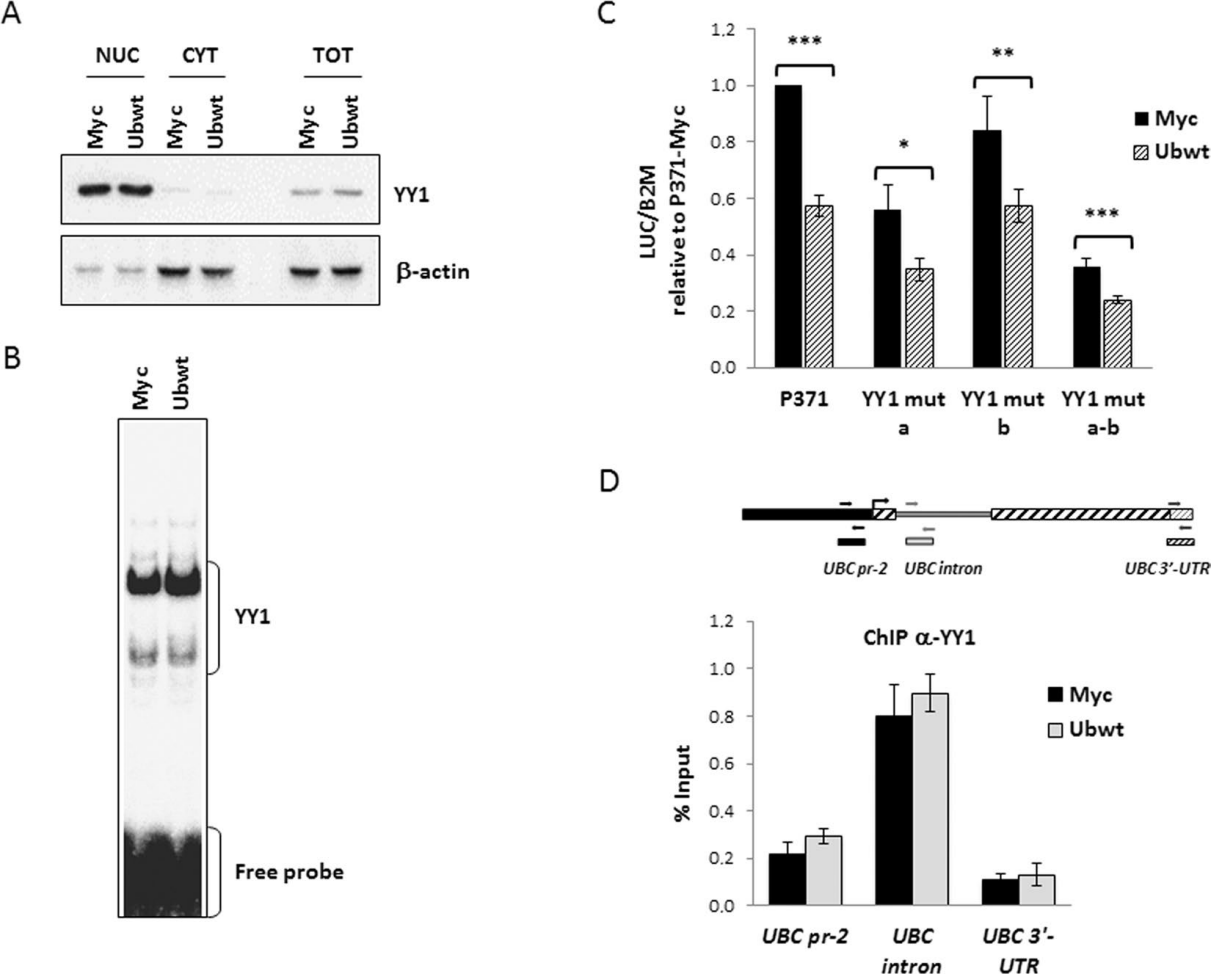
Investigating the role of YY1 transcription factor in *UBC* downregulation. (**A**) Immunoblot analysis of YY1 protein factor in nuclear, cytosolic and whole extracts obtained from HeLa cells transiently transfected with the Ub expression vector (Ubwt) or the empty control vector (Myc). β-actin was used as loading control. (**B**) EMSA analysis was performed using a ^32^P-labeled oligonucleotide containing the most upstream YY1 binding site previously identified in the *UBC* intron region^39^, as probe. Protein/DNA complexes were detected upon incubation of probe with nuclear extracts (5 μg) from cells transfected as in (**A**). (**C**) The wild-type reporter construct P371 and the YY1 mutant counterparts (YY1 mut a, b, a-b) were cotransfected in HeLa cells with the Ub expression construct or the empty vector pCMV-Myc. Forty-eight hours post-transfection, luciferase mRNA levels were determined, normalized to *B2M* and expressed as fold change versus the P371/Myc cotransfected sample, set to 1 (n = 6). (**D**) ChIP analysis of Myc and Ubwt transfected cells, using an antibody specific for YY1; for the internal IP control, no Ab was added. Detection of ChIPed DNA and data analysis were as described in the legend of Fig. 4C (n = 2, assayed in duplicate). Positions of the primers used are depicted in the scheme above the histogram. Data presented are means ± SEM from the indicated number of experiments. Asterisks indicate statistical significance: *p < 0.05; **p < 0.01; ***p < 0.001 vs. control (Myc). The images shown in (**A**,**B**) are representative of three independent experiments. Full-length immunoblots and EMSA are presented in Supplementary Fig. S8.

We previously demonstrated that YY1 positively regulates *UBC* gene expression in basal conditions, by interacting with two intronic binding sites^39^. Based on this finding and on the evidence that ubiquitin-proteasome mediated degradation of YY1 represents a post-translational regulatory mechanism for this protein factor^47^, we examined the possible effects of ubiquitin overexpression on YY1 protein levels and/or intracellular distribution. Western blot analysis of whole cell lysates, as well as of nuclear and cytosolic extracts obtained from pCMV-Myc and Ubwt transfected cells showed that total YY1 content did not change upon Ub transfection and there was no difference in the amount of YY1 in either the cytosolic and nuclear fractions (Fig. 6A). Next, we examined whether increasing Ub pools affected the DNA binding activity of YY1; however, the EMSA performed with a probe containing the most upstream intronic YY1 binding site, revealed a comparable pattern of retarded bands of similar intensities, in control and ubiquitin overexpressing cells (Fig. 6B). Next, we investigated the effects of Ub overexpression on the transcriptional activity of different reporter constructs (depicted in Supplementary Fig. S2B) where luciferase expression is driven by the *UBC* promoter fragment, referred to as P371, harboring wild-type or mutagenized YY1 binding sites. The results shown in Fig. 6C demonstrated that the mutant constructs behaved the same as the wild-type construct, that is luciferase mRNA significantly decreased after ubiquitin transfection, suggesting that YY1 may not be involved in the downregulation effect. Further confirmation has been obtained by the *in vivo* ChIP assay showing that YY1 occupancy of the *UBC* promoter region did not change upon ubiquitin overexpression (Fig. 6D). A similar percent input was indeed detected for pCMV-Myc and Ub transfected cells when ChIPed DNA was amplified with primers encompassing the main YY1 binding site in the intron (*UBC* intron), the YY1 motif detected in the proximal promoter region and previously proved to be not functional (*UBC* pr-2) and a negative control sequence (*UBC* 3′-UTR).

### H2A and H2B histone ubiquitination signatures of *UBC* promoter do not change upon ubiquitin overexpression

**Figure 7:**
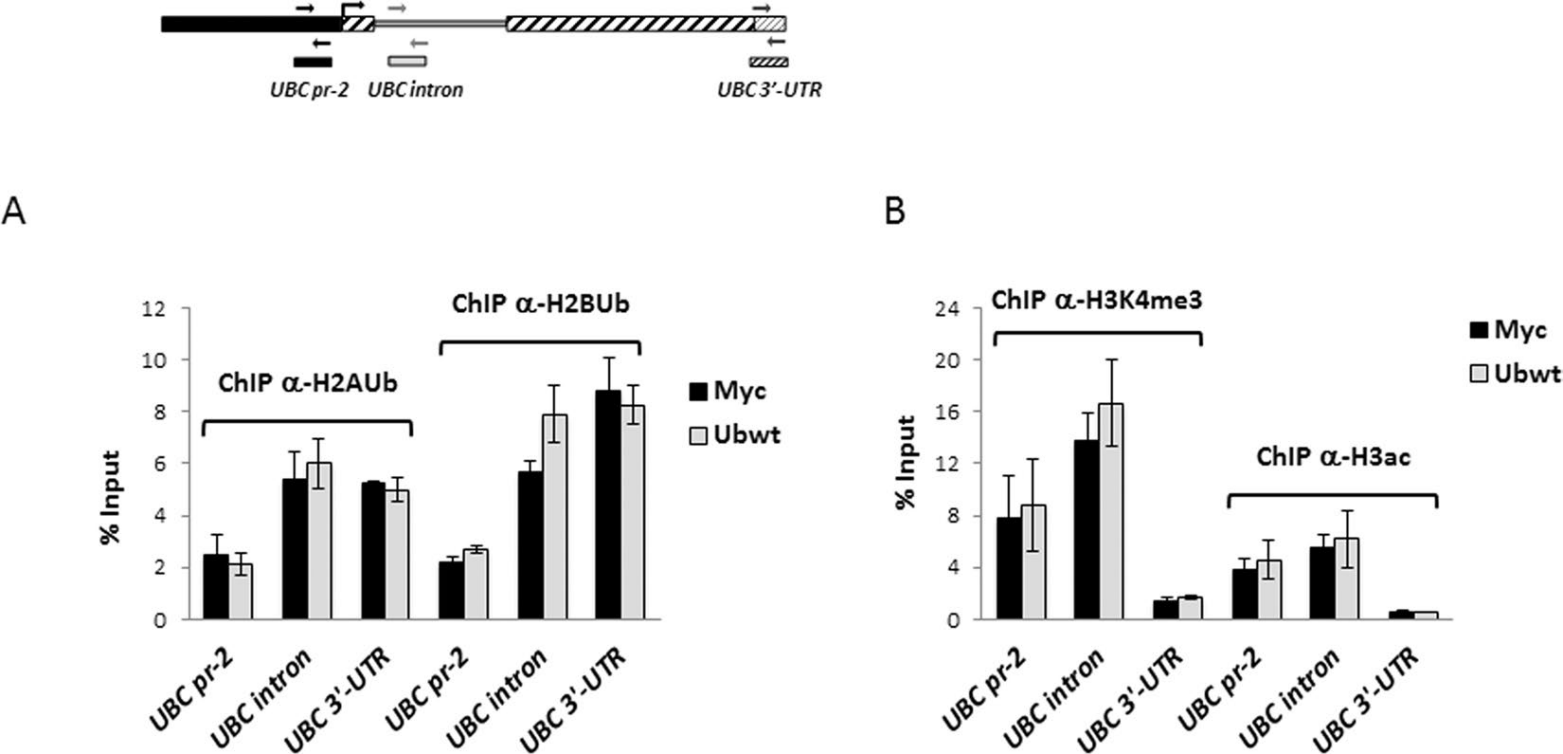
Investigating the role of histone modifications in *UBC* downregulation. (**A**,**B**) Chromatin immunoprecipitation experiments on HeLa cells transfected with the Ub expression vector (Ubwt) or the empty control vector (Myc). Cells were harvested 48 h post transfection and ChIP was performed, as detailed in Materials and Methods, using antibodies specific for H2AUb and H2BUb (**A**) or for H3K4me3 and H3ac (**B**). The scheme on top shows the location of the primers used in RealTime PCR. Results are expressed as % of chromatin input controls. The histograms show the mean ± SEM of two independent experiments, analyzed in duplicate. No statistical significance was detected in Ubwt versus Myc control sample.

Ubiquitination of histone proteins (mainly of H2A and H2B) has been characterized as an important epigenetic mechanism regulating gene transcription. In particular, H2AK119ub functions as a transcriptional repressor^48,49^, while H2BK120ub in general promotes gene transcription, by different mechanisms, like favoring H3K4 methylation^50,51^. Therefore, we sought to investigate if ubiquitin overexpression could alter the ubiquitination status of the *UBC* promoter region, thus accounting for the lowered *UBC* mRNA levels detected upon Ub transfection. We performed chromatin immunoprecipitation on cells transfected with Ub or the control empty vector, using antibodies specific for monoubiquitinated histones H2A and H2B, followed by amplification of different promoter regions. No significant differences were detected in the percent input obtained for control (Myc) and ubiquitin overexpressing (Ubwt) cells (Fig. 7A). Likewise, chromatin immunoprecipitation with anti-H3K4me3 and anti-H3ac (both marks of transcriptionally active chromatin) did not show significant differences between Myc and Ub samples (Fig. 7B).

### Ubiquitin overexpression does not impact the transcriptional activity of the *UBC* promoter, but rather the splicing of nascent *UBC* transcripts

**Figure 8:**
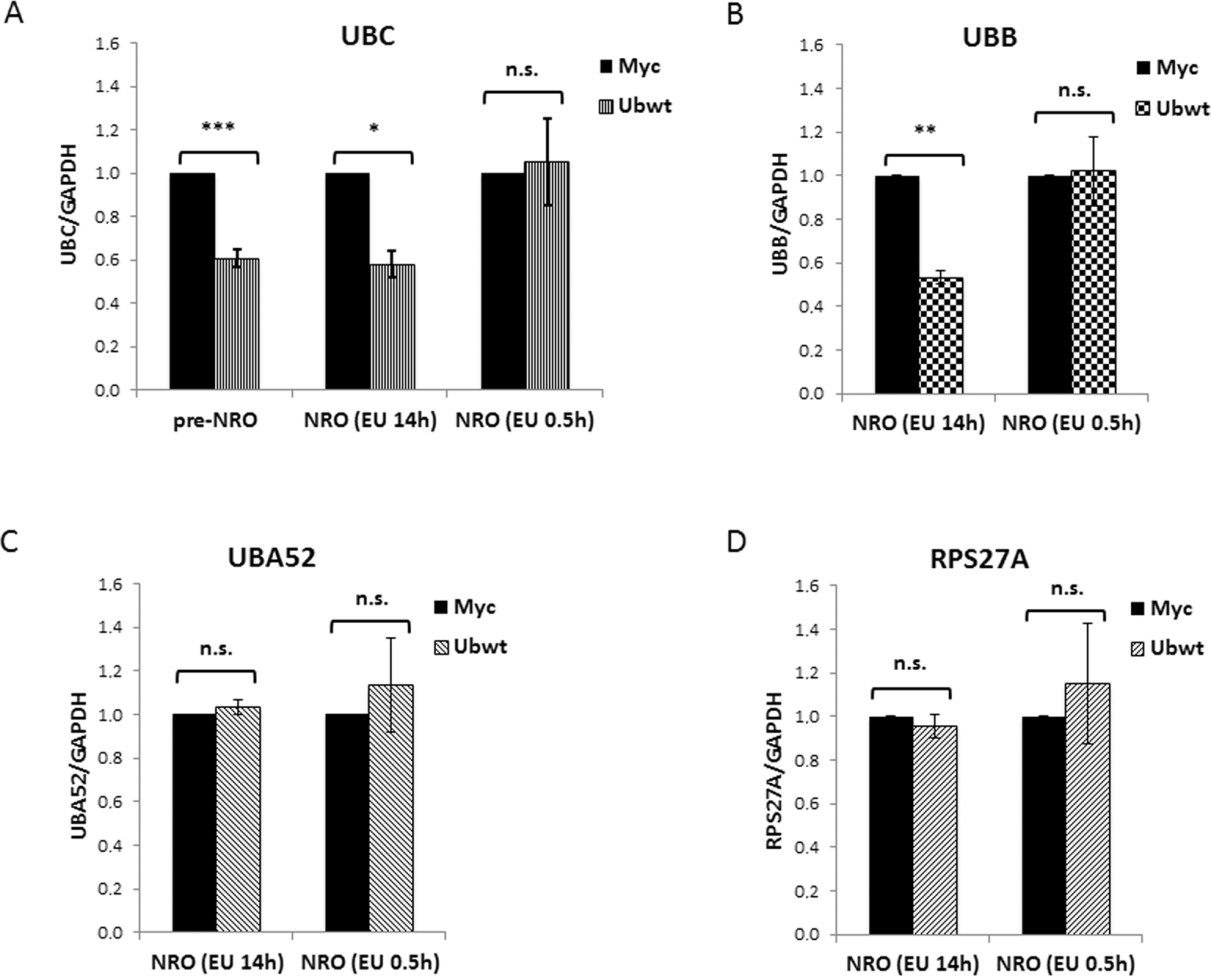
Metabolic labeling of nascent transcripts. HeLa cells transfected with the Ubwt expression construct or the empty vector pCMV-Myc were pulsed (48 h post-transfection) either with 0.5 mM 5-ethynyl Uridine for 0.5 hours (NRO, EU 0.5 h) or with 0.2 mM 5-ethynyl Uridine overnight (NRO, EU 14 h). Cells were then collected and processed essentially following the protocol provided with the Click-iT Nascent RNA Capture kit (Thermo Fisher Scientific). (**A**) RTqPCR analysis of nascent *UBC* transcripts in transfected HeLa cells, pulsed as described above. For comparison, the RTqPCR performed on total RNA extracted from unpulsed Myc and Ub transfected cells is shown in the histogram (pre-NRO). (**B**–**D**) RTqPCR analysis of *UBB* (**B**), *UBA52* (**C**) and *RPS27A* (**D**) nascent transcripts in transfected HeLa cells, pulsed as above. The values of RT-qPCR shown are mean ± SEM of five independent experiments assayed in duplicate. Statistical significance: *p < 0.05; **p < 0.01; ***p < 0.001 vs. control (Myc); n.s., not significant.

**Figure 9:**
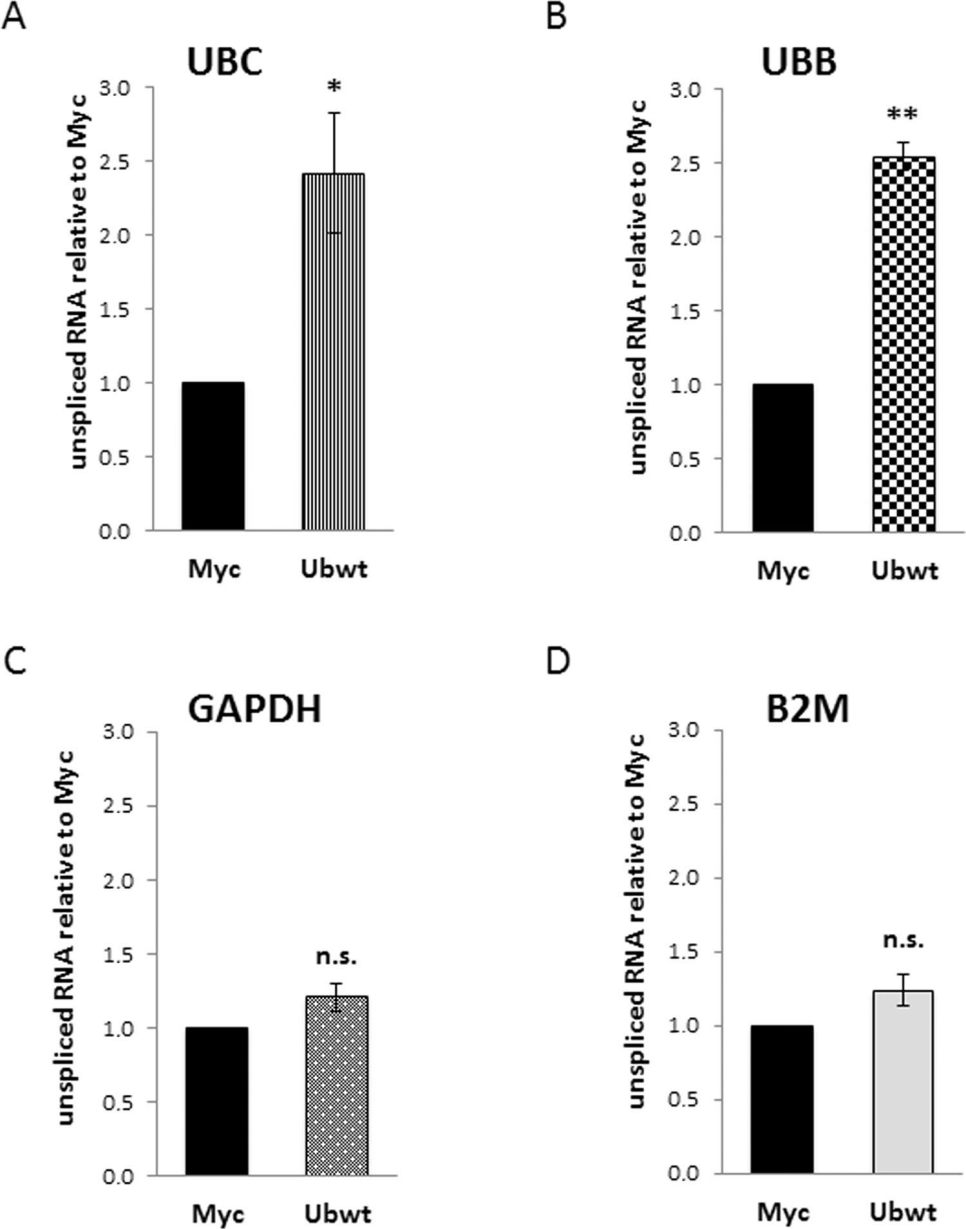
Analysis of unspliced transcripts in the nuclear fractions. (**A**–**D**) RTqPCR analysis of unspliced transcripts in the nuclear RNA samples extracted from Myc and Ub-transfected cells. Each target gene was analyzed with both primers amplifying total mRNA and primers detecting only the unspliced transcript. The fraction of unspliced transcripts in Myc and Ub samples was calculated with the 2^−ΔCt^ formula (where ΔCt means Ct_unspliced mRNA_-Ct_total mRNA_). Graphs show the fold change of unspliced mRNA in Ubwt versus Myc, set to 1. The values of RTqPCR data shown in (**A**–**D**) are the average ± SEM of four independent experiments analyzed in duplicate. Statistical significance: *p < 0.05; **p < 0.01 vs. control (Myc); n.s., not significant.

Having not found the *cis-trans* elements involved in the *UBC* downregulation during Ub overexpression, we measured the *UBC* transcriptional activity using the run-on assay. This approach relies on the quantification of biochemically labeled nascent RNA molecules by RTqPCR. Transfected cells were pulsed with 0.2 mM and 0.5 mM 5-ethynyl Uridine, respectively for 14 h and 0.5 h. RTqPCR analysis of nascent *UBC* transcripts showed a ubiquitin-dependent downmodulation of *UBC* (similar to the one detected by RTqPCR of “steady-state” RNA samples) when pulse labeling was left overnight, but no difference in the transcription rate was found after 0.5 h pulse labeling (Fig. 8A). The same layout has been observed for *UBB*, the other ubiquitin coding gene repressed by ubiquitin overexpression (Fig. 8B), while the absence of downregulation was reconfirmed for the Ub-ribosomal fusion genes *UBA52* and *RPS27A*, in both pulse labeling conditions (Fig. 8C,D). These results prompted us to investigate a post-transcriptional mechanism accounting for ubiquitin overexpression-dependent *UBC* downregulation. Thus, we focused on the splicing process to see if ubiquitin overexpression could in some way affect the splicing efficiency of the unique intron of the *UBC* gene. The first stage was the preparation of intact nuclei from Myc and Ub-transfected cells to measure the amount of unspliced transcripts within the pool enriched of nascent RNAs. Different assays were performed to check the fidelity of nuclei harvesting and proper separation of the cytosolic fraction: intact nuclei were visualized by light microscopy and compared with whole cells (Supplementary Fig. S3A); western blot analysis of protein expression in the purified nuclei revealed the presence of lamin A/C (as expected) but the absence of GAPDH, which is a typical cytoplasmic marker (Supplementary Fig. S3B). RNA samples purified from the nuclear and cytosolic fractions were analyzed by denaturing agarose gel electrophoresis, in parallel with a total RNA sample extracted from whole cells. As shown in the Supplementary Fig. S3C, the ribosomal RNAs (18 S and 28 S) were predominant in the cytosol compared to the nuclear fraction, in agreement with their shuttling to the cytosol upon maturation and assembly with the ribosomal proteins. The nuclear total RNA samples were subjected to retrotranscription followed by RTqPCR, performed both with primers expressly designed to detect the unspliced transcripts, and with standard primers used to measure total mRNAs (Supplementary Table S1). In the ubiquitin overexpressing cells, the fraction of unspliced *UBC* mRNA was about 2.4-fold higher compared to pCMV-Myc transfected cells (Fig. 9A); the same occurred for the *UBB* RNA (2.5-fold more unspliced transcript in Ubwt vs Myc; Fig. 9B); while the percentage of immature nascent RNAs, in Myc and Ub samples, was not statistically different for the housekeeping genes *GAPDH* (Fig. 9C) and *B2M* (Fig. 9D) (∼1.2-fold in Ubwt vs. Myc sample for both targets). The same analysis performed on cytoplasmatic RNAs did not detect any difference in the residual unspliced transcripts between Myc and Ub receiving cells, for all the targets investigated.

Finally, we sought to investigate the stability of *UBC* mRNA in a steady state, after inhibition of *the novo* transcription by Actinomycin D, which blocks RNA polymerases. HeLa cells transfected with Myc and Ubwt expression vectors were treated with ActD, harvested at 5 time points (0, 1, 2, 3 and 4 h) after ActD treatment, and then analyzed by RTqPCR. The estimated half-lives of *UBC* mRNA were not statistically different in Myc and Ubwt samples (1.28 and 1.21 h, respectively) (Supplementary Fig. S4).

## Discussion

Ubiquitin coding genes serve housekeeping functions providing cells with adequate levels of free ubiquitin required to sustain homeostasis. We previously reported that the polyubiquitin gene *UBC* mainly contributes to the intracellular ubiquitin content under basal conditions^52^; moreover, it is at the frontline to supply the extra-ubiquitin needed upon cell exposure to a variety of stresses^9,16,42,52,53^. The ability to buffer different cellular needs requires a highly dynamic but strict regulation of the *UBC* gene^54^. The molecular bases of *UBC* induction, when cells demand more Ub, have been extensively investigated; less known are the consequences of raising the intracellular Ub levels on *UBC* gene expression. In this study, we generated Ub overexpressing cells, in which both the free and conjugated Ub pools proportionally increased^28^, demonstrating that the exogenous ubiquitin is properly processed and channeled into the pathways to be used as a signaling tag. Furthermore, Ub overexpression led to a significant decrease in the Ub expression from the two polyubiquitin genes *UBB* and *UBC*. To the best of our knowledge, a similar evidence has only been described by the S. M. Wilson team in transgenic mice that expressed a HA-poly-Ub, under the control of a neuronal promoter^25,27^. The authors highlighted the selective sensitivity of neurons towards minimal changes in the Ub pool, which hence requires a strict control of Ub homeostasis in the neuronal system, but did not investigate the molecular mechanisms underlining this behavior. These latter became the focus of this study. Firstly, the evidence that downregulation of *UBC* occurred in different cell lines, both normal and tumor-derived, suggested that it might constitute a widespread regulatory response to Ub overexpression. Secondly, transfection of different amounts of Ub expression plasmid allowed to raise the ubiquitin levels without dramatically overexpressing it, thus enhancing the chance of identifying regulatory pathways that operate in a physiological fluctuation range of Ub and not in the context of a ubiquitin stress response^21^. Doubling the total Ub pool is sufficient to significantly downregulate *UBC* in HeLa cells. Thus, the downmodulation of *UBC* may be part of an (auto)regulatory feedback loop through which ubiquitin attempts to maintain its cellular homeostasis. This entails the existence of a sensing mechanism capable to detect the ubiquitin levels and consequently modulate the transcriptional programs of the polyubiquitin gene *UBC*. This study aimed to identify this putative Ub sensor, as well as the relevant *cis*-elements in the *UBC* promoter engaged in downmodulation. Actually, the Ub sensor could act by binding non-covalently to Ub or by direct conjugation to Ub itself. To dissect the “language” of ubiquitin sensing, we took advantage of several Ub analogues. The data obtained suggest that Ub sensing requires ubiquitin conjugation, since transfection of Ub mutants devoid of a wild-type C-terminus (UbG76A and particularly UbΔGG) does not cause *UBC* downregulation. Among the lysine mutants, only UbK0, an engineered form of ubiquitin in which all seven lysine residues are replaced with arginine^55^, failed to induce the downmodulation of *UBC* gene, when overexpressed in HeLa cells, suggesting that the assembly of a poly-Ub chain not linked via K48 or K63 (excluded by the K48R and K63R Ub mutants) might be necessary for downregulation to occur. Although the use of Ub analogues, mainly of those highly mutagenized, has been debated, Huang *et al*.^56^ demonstrated that K0-Ub adopts the same backbone structure as wild-type ubiquitin and, what is more important, it is readily managed by the enzymes E1 and E2.

On the whole, Ub analogues indicated that the “cellular component” in charge of Ub sensing relies on a direct Ub conjugation event, feeding the attractive hypothesis that the “excess” of ubiquitin could promote the destruction or inactivation of protein(s) involved in *UBC* gene transcription, thus providing a straightforward negative feedback loop responsive to intracellular ubiquitin levels.

Previous studies by our group^38,39^ and others^43,53^ have partially dissected the transcriptional regulation of *UBC* promoter defining the relevant *trans-*acting factors responsible for basal and stress-inducible gene expression. When the luciferase reporter vector P916 (containing the *UBC* promoter region spanning from 916 nt upstream of the TSS to the end of the intron) was cotransfected in HeLa cells with the Ub expression construct, a decreased luciferase transcription was detected, suggesting that the regulatory button might be contained within this sequence. *UBC* has long been labeled as “stress-responsive gene” and HSF1 and HSF2 transcription factors have been demonstrated to be the key regulators of *UBC* induction under different stressful conditions. Although the mechanism of their activation is different^57^, both HSFs undergo ubiquitin and proteasome dependent degradation^44–46^. Therefore, we sought to investigate if overexpression of ubiquitin affected their levels and/or DNA binding activity: we didn’t find any difference in Ub overexpressing cells compared to pCMV-Myc transfected cells. But, when cells transfected with the Ubwt expression vector were treated with the proteasome inhibitor (MG132), they activated the HSR, as demonstrated by the HSF1-dependent expression of *HSP70*. Strangely enough, in these conditions, they also induced *UBC* gene transcription, despite the abundant cellular Ub content. This may be explained by the activation mechanism of HSF1, requiring its dissociation from the inhibitory partners, namely chaperones, which are displaced by misfolded proteins accumulating under proteotoxic stress environment^44,58^. Free HSF1 acquires competence to bind the HSEs in the promoter of target genes, inducing their transcription. For *UBC*, this occurs independently of the high cellular Ub content, since HSF1 does not sense the ubiquitin levels, but the presence of unfolded proteins^58,59^. It is intriguing that the *UBC* promoter can be modulated by and respond to opposing mechanisms at the same time: the downmodulation driven by the high Ub levels on one side, and the HSF-mediated upregulation triggered by the induced proteotoxic stress on the other side. The molecular bases of this regulatory crossroad and the seemingly contradictory behavior of the *UBC* gene certainly deserve further investigations.

Data obtained by cotransfection of reporter constructs with the Ub expression vector provided compelling *in vivo* evidence that the *UBC* promoter region warranting the downregulation effect requires the presence of the intron and of an upstream promoter sequence of at least 123 nt, although the essential *cis*-elements for a strong basal expression are comprised within the −371/+876 spanning sequence^38,43^. This region harbors different binding sites for the ubiquitous TF specificity protein 1 (Sp1), which has been proved to be important for the basal expression of *UBC* gene^38,43^. Moreover, it is known that Sp1 undergoes ubiquitination-dependent proteasomal degradation^60,61^. However, its participation to *UBC* downregulation has been ruled out by different *in vitro* and *in vivo* experiments.

Intron removal from the luciferase reporter constructs abolished the downregulation effect, so we turned our attention towards YY1, the main TF previously found to sustain basal *UBC* gene expression by interacting with multiple binding sites in the intron sequence^39^. Using various *in vitro* experimental approaches, we demonstrated that Ub overexpression did not alter either YY1 protein levels and intracellular distribution or its DNA binding activity. Moreover, *in vivo* studies confirmed that YY1 occupancy of the *UBC* promoter was unchanged in Ub transfected cells and, according to this evidence, mutagenesis of the intronic YY1 binding motifs in the reporter construct did not impair the downregulation mechanism.

The hitherto reported evidences point towards a different kind of contribution of the intron to the downregulation effect, beyond the interaction with transcriptional regulators: it might for example undergo conformational changes, upon Ub transfection, that negatively affect the assembly and/or activity of the transcriptional machinery. Histone ubiquitination is an important epigenetic mechanism for regulating chromatin structure and ultimately gene transcription. Mono-ubiquitination of histone H2A (H2AUb) is a reversible transcriptionally repressive mark^49^, while H2B ubiquitylation (H2BUb), interferes with chromatin compaction and leads to an open and more accessible conformation, thus favoring transcription^50^. In addition, depletion of ubiquitylated histone H2A has been detected during proteotoxic stress, to support the accumulation of Ub conjugates, meaning that a redistribution of ubiquitin between different “Ub demanding substrates” indeed exists^19,62,63^. Remarkably, we found no changes in the ubiquitination of histones H2A and H2B within the *UBC* promoter region upon ubiquitin overexpression, meaning that likely it is not a change in histone ubiquitylation responsible for the *UBC* downregulation.

Taken together, the evidences so far described show that the main protein factors related to gene transcription do not seem to be involved in the downregulation and the chromatin landscape around the *UBC* promoter region doesn’t exhibit noteworthy signatures upon Ub overexpression. Therefore, we wondered whether the higher Ub levels actually dampened *UBC* gene expression, by acting at transcriptional level. To investigate the transcription initiation step, we detected the nascent *UBC* transcripts using the Click-iT technology, which facilitates the partitioning of the newly synthesized RNA transcripts from the already existing RNAs, without the need to isolate the nuclei, as in the classical run-on assay. Of note, results of RT-qPCR analysis of nascent transcripts, upon short pulse labeling with 5-ethynyl uridine, showed that Ub overexpression did not produce significant changes in *UBC* transcription. On the contrary, when labeling with EU was maintained for longer time, a downregulation similar to that found for “steady-state” RNA samples was detected. While the first condition allowed the determination of the actual frequency of generation of nascent transcripts (i.e., the transcription initiation rate), the longer time pulse measured the steady state levels of mRNAs which, besides the “real” promoter activity, depend on several post-transcriptional events. Analysis of nascent transcripts from the *UBB* gene, which was found downregulated upon exogenous Ub expression like *UBC*, produced the same output as *UBC*. Information gained by nascent transcript analysis excludes a transcription rate control, while results from reporter constructs highlight the requirement of the endogenous intron for the *UBC* gene response: on the whole, these evidences put forward the idea that the downregulation effect might arise in the co- or post-transcriptional phase. This was assessed by determining the levels of unspliced mRNAs in the pool of transcripts purified from the nuclear fractions. For *UBC*, the unspliced pre-mRNAs were significantly increased in Ub overexpressing cells relative to cells transfected with the empty vector. Of note *UBB* showed an increase of the unspliced fraction like *UBC*, while the housekeeping genes *GAPDH* and *B2M* were unaffected by the higher ubiquitin levels, suggesting that the effect is specific. Notably, from studies on reporter constructs we found out that the replacement of the endogenous *UBC* intron with a “splicing-competent” heterologous intron sequence did not support the downregulation following Ub overexpression, indicating that, in addition to splicing sites, other *cis*-*trans* acting elements specific to the *UBC* intron are strictly required for downregulation to occur. Regarding the *UBB* gene, which exhibited a comparable change on splicing efficiency like *UBC*, in silico analyses showed that human *UBB* and *UBC* introns do not share much similarity (around 47%). Moreover, searching for transcription factor binding sites, using two bioinformatic tools, ALGGEN (http://alggen.lsi.upc.es/cgi-bin/promo_v3/promo/promoinit.cgi?dirDB=TF_8.3) and TFBIND (http://tfbind.hgc.jp/) found different putative YY1 binding sites within *UBB* intron. This is quite intriguing and certainly deserves further investigation, although YY1 seems to not participate to the ubiquitin mediated *UBC* downmodulation.

The finding that increased Ub levels impaired splicing of the *UBC* transcript may represent one of the mechanisms leading to *UBC* downmodulation. The involvement of ubiquitination/deubiquitination cycles in the regulation of co-transcriptional splicing has recently been documented^64–66^. Milligan *et al*. reported that reversible ubiquitination of the catalytic subunit of RNA polymerase II is required to induce a slowed elongation rate (transcriptional pausing), thus favoring co-transcriptional splicing, and is then removed by a protease complex associated with the nascent transcript^64^. Moreover, cells make a large use of ubiquitin and ubiquitin-like proteins (UBLs) to modify (either covalently or non covalently) different spliceosomal components, in order to modulate spliceosome assembly, to control splicing fidelity and to fine-tune the process of pre-mRNA splicing (reviewed in^65^). Of particular interest is the role of the UBL Sde2, a ubiquitin-fold containing splicing regulator that confers intron specificity to the spliceosome^65,66^. The Sde2 precursor undergoes a ubiquitin-like processing, mediated by DUBs, to be activated and then incorporated into the spliceosome, to promote splicing of selected introns from a subset of pre-mRNAs. Moreover, the N-terminal lysine residue of processed Sde2 makes it a short-lived protein being a good substrate of the N-end rule pathway of proteasomal degradation^66^. Based on these evidences, it would be exciting to investigate if the elevated ubiquitin levels affect the post-translational modifications of spliceosomal components and/or the abundance of intron-specific splicing regulators, similar to Sde2.

To summarize results herein presented, under Ub overexpression background, the mature *UBC* mRNA levels significantly decrease, while the fraction of unspliced *UBC* pre-mRNA increases. Having excluded a transcriptional control, we speculate that the inefficient *UBC* intron splicing generates a higher amount of intron-retaining transcripts, which are quickly degraded by the cell to prevent the expression from these nonfunctional RNAs^67–69^ and this results in lower *UBC* gene expression.

We previously demonstrated and investigated at molecular level the role of the intron in basal *UBC* expression; herein we found evidence that efficient intron splicing may be affected by the cellular ubiquitin levels, thus providing an additional layer of regulation of the *UBC* gene, and a cellular strategy to control Ub pool homeostasis. Additional work is needed to dissect how ubiquitin and/or its sensor(s) participate to modulate this activity.

## Materials and Methods

### Cell lines and treatments

Mammalian cell lines used in this study were the cervical carcinoma HeLa cells, the osteosarcoma U2OS cells, the human embryonic kidney 293 (HEK293) cells, all obtained from American Type Culture Collection (ATCC, Manassas, VA, USA) and the normal human keratinocytes NCTC-2544, obtained from Interlab Cell Line Collection (ICLC, Genova, Italy). HeLa cells were maintained in Roswell Park Memorial Institute (RPMI) 1640 medium containing 10% fetal bovine serum, 100 U/mL penicillin and 100 μg/mL streptomycin, while the other cell lines were maintained in Dulbecco’s Modified Eagle Medium (DMEM) supplemented as above, with the addition of 0.1 mM nonessential amino acids for HEK293. All the cell lines were grown at 37 °C and proved to be mycoplasma-free using EZ-PCR Mycoplasma Test Kit (BI, Biological Industries). Two days post-transfection, cells were treated with 20 μM proteasome inhibitor MG132 (Selleckchem, Munich, Germany) or the vehicle DMSO as a control, for 4 h at 37 °C, and then harvested for gene expression analyses. To measure mRNA half-life, at day 2 post-transfection of HeLa cells with the ubiquitin expression construct, 2.5 μg/mL Actinomycin D (ActD; Sigma, St. Louis, MO, USA) was added to cells to inhibit transcription. Cells receiving the vehicle DMSO were set up as control. After 0, 1, 2, 3 and 4 h of ActD treatment, cells were collected, RNA was extracted, and qPCR was carried out with primers used to measure total mRNAs.

### Plasmid constructs and transfections

The preparation of the expression constructs for wild-type ubiquitin and for the lysine mutants K48R and K63R has been described elsewhere^28^, while the ubiquitin mutant UbG76A has been obtained as detailed in^33^. The wild-type and mutant Ub-Myc coding sequences were released from the resident plasmid by Pst I/Klenow and Kpn I treatment and cloned into the pCMV-Myc vector between the Apa I/Klenow and Kpn I sites. The plasmid encoding for the I44A ubiquitin has been obtained using the Quick-Change Site-Directed Mutagenesis kit (Stratagene Inc., La Jolla, CA), with the wild-type construct as template, and the degenerate primers designed to introduce the selected amino acid substitution, shown in the Supplementary Table S1. To generate the recombinant vector for the expression of an ubiquitin molecule lacking the two carboxy-terminal glycines (UbΔGG), the construct bearing the wild-type Ub coding sequence was used as a template in a PCR reaction, performed with the Platinum Pfx DNA polymerase (Invitrogen, Carlsbad, CA, USA), according to the manufacturing instructions, and the degenerate primers shown in the Supplementary Table S1. The forward primer was engineered to be cut with Apa I restriction enzyme, while the reverse primer, bearing a Kpn I cutting site, was designed to allow the deletion of the glycine codons at positions 75 and 76, and to provide a translation stop codon to the insert. The PCR product, Apa I/Kpn I digested, was then inserted into the pCMV-Myc vector (Clontech, Mountain View, CA), cut with the same restriction enzymes. DNA encoding for a Lys-less mutant ubiquitin (UbK0), an engineered form of ubiquitin in which all seven lysine residues are replaced with arginine, has been obtained from the pRK5-HA-Ubiquitin-K0, which was a gift from Ted Dawson (Addgene plasmid # 17603; http://n2t.net/addgene:17603; RRID:Addgene_17603)^70^. Forward primer containing an Apa I restriction site and reverse primer with a Kpn I restriction site were used to amplify the UbK0 coding sequence, which was cloned into the pCMV-Myc. The UbK0 expression vector underwent a mutagenesis reaction to put a Kozak sequence just upstream of the translation initiation codon, using the primer reported in the Supplementary Table S1.

Most of the 5′- and 3′-serially deleted reporter constructs for the *UBC* promoter study have been previously developed^38,39^. New reporter constructs were generated during the present study with four different forward primers (named, respectively, −254, −195, −123 and −84 according to their upstream position relative to the TSS, set to +1), all bearing a Sac I cutting site and a common reverse primer, carrying a Hind III cutting site, positioned 876 nt downstream of the transcription start site, at the end of the *UBC* intron (see Supplementary Table S1). The single-site and multi-site mutations of Sp1 and YY1 transcription factor binding motifs lying in the intron sequence, were all performed using the reporter construct carrying the −371/+876 *UBC* promoter region (P371, previously referred to as P3), as template^39^. The mutagenesis of the HSEs in the untranscribed region of the longer promoter construct (P916, previously named P1) to obtain the HSF mutants has been already described^42^. All DNA sequences were validated and confirmed through DNA sequencing using a PE310 Perkin Elmer capillary sequencer. The plasmid constructs used in this study are listed in the Supplementary Table S2. Plasmid DNA for mammalian cell transfection was propagated in XL1-Blue or NovaBlue bacterial strains and purified by EndoFree Plasmid Maxi Kit (Qiagen Inc., Valencia, CA, USA).

HeLa and U2OS cells were transiently transfected using Effectene Transfection Reagent (Qiagen), while HEK293 and NCTC-2544 were transfected using the GeneCellin reagent (BioCellChallenge SAS, France), following the protocols’ instructions. Healthy cells were seeded the day before transfection in 6-well plates in order to reach ~60–70% confluence at the time of transfection with 0.4 µg (Effectene) or 2 µg (GeneCellin) plasmid DNA/well, respectively. When HeLa cells were transfected with increasing amounts of Ubwt expression plasmid, the total amount of transfected DNA was kept constant at 0.4 μg by compensating with the pCMV-Myc vector. In cotransfection experiments, 0.2 µg of luciferase reporter plasmid and 0.4 µg of Ub expression vector or empty vector pCMV-Myc, were added to each well. Cells were harvested 2 days after transfection for luciferase assay, RNA and protein analyses.

### Luciferase reporter assay

Forty-eight hours post-transfection cells were treated with lysing buffer and luciferase activity was determined by the Luciferase Assay Reagent (Promega s.r.l., Milano, Italia) according to the manufacturer’s protocol, on a FLUOstar OPTIMA multifunction microplate reader (BMG-LABTECH GmbH). The firefly luciferase activity was normalized against total protein concentration, as previously reported^38^.

### RNA preparation and quantitative real-time RT-PCR (RTqPCR)

RNA was extracted from transfected cells by using the RNeasy Plus Mini kit (Qiagen) according to the manufacturer´s instructions, followed by DNase treatment (with the TURBO DNA-free™ Kit from Ambion, Austin, TX) to remove any traces of plasmid DNA, when cells received luciferase constructs. RNA concentration was measured by Nanodrop ND-1000 System (NanoDrop Technologies, Wilmington, DE). cDNA for all samples was prepared from 500 ng of total RNA using PrimeScript^TM^ RT Master Mix (Perfect Real Time; Takara Bio Europe SAS, Saint-Germain-en-Laye, France). SYBR green based Real-Time PCR was performed with Hot-Rescue Real Time PCR Kit (Diatheva s.r.l., Cartoceto PU, Italy), essentially according to the manufacturer’s instructions, using an ABI PRISM 7700 Sequence detection system (Applied Biosystems, Foster City, CA, USA)^16^. Thermal cycling was performed as follows: 10 min at 95 °C; 40 cycles of denaturation at 95 °C for 15 s, annealing at 60 °C for 15 s, and extension at 72 °C for 30 s. At the end of PCR cycles, a melting curve was generated to verify the specificity of PCR products. All measurements were performed in triplicate and reported as the average values ± standard error of the mean (mean ± SEM). Target gene values were normalized with the housekeeping genes glyceraldehyde 3-phosphate dehydrogenase (*GAPDH*) or beta 2-microglobulin (*B2M*), as specified. Expression data were calculated according to the 2^−ΔΔCt^ method^71^. Sequences of primers used for RTqPCR are reported in the Supplementary Table S1.

### Cell extracts

For protein expression analysis, cells harvested 48 h post-transfection were washed in PBS and lysed by sonication in sodium dodecyl sulfate (SDS) buffer containing 50 mM Tris/HCl pH 8.0, 2% (w/v) SDS, 10 mM N-ethylmaleimide supplemented with a cocktail of protease inhibitors (Roche Diagnostics, Mannheim, Germany). Lysates were boiled and then cleared by centrifugation at 12,000 × *g*. Protein concentration was determined according to Lowry, using bovine serum albumin as standard. Nuclear extracts were obtained by low salt/detergent cell lysis followed by high salt extraction of nuclei as previously described^16^ and protein concentration was determined by the Bradford assay.

### Western blotting

For Western blot analysis, protein samples were separated by SDS-PAGE, transferred to a nitrocellulose membrane (0.2 mm pore size; Bio-Rad, Hercules, CA, USA). The blots were probed with the primary antibodies listed below and bands were detected by horseradish peroxidase (HRP)-conjugated secondary antibody (Bio-Rad). Peroxidase activity was detected with the enhanced chemiluminescence detection method (WesternBright ECL, Advasta, Menlo Park, CA, USA). Primary antibodies used in this study were: rabbit polyclonal anti-ubiquitin antibody (kindly provided by Prof. A. L. Haas, Dept. of Biochemistry and Molecular Biology, Louisiana State University Health Sciences Center, New Orleans); anti-HSF2 (sc-13056), anti-YY1 (sc-281), anti-specificity protein 1 (Sp1) (sc-420) and anti-specificity protein 3 (Sp3) (sc-644) from Santa Cruz Biotechnology (Dallas, TX, USA); anti-Lamin A/C (4C11) and anti-HSF1 (4356) from Cell Signaling Technology (Danvers, MA, USA); anti-GAPDH (A300-641A) from Bethyl Laboratories, Inc.; anti-actin (A 2066) from Sigma-Aldrich (Steinheim, Germany).

### USP2 digestion and solid phase immunoassay

For quantification of total Ub levels, cells were washed with ice-cold PBS and lysed by sonication (4 cycles of 15 sec at 25 Watt) in 50 mM Na_2_HPO_4_/NaH_2_PO_4_ pH 7.4, 140 mM NaCl, 2 mM β-mercaptoethanol supplemented with protease inhibitors. Cell extracts were cleared by centrifugation and protein content was determined by the method of Bradford. Twenty µg of extract were incubated at 37 °C for 60 min with 0.5 µg of human recombinant Usp2 protein in a final volume of 40 μL. The digestions were stopped by adding an equal volume of SDS-PAGE sample buffer and boiling. Conversion of polymeric Ub to Ub monomers was checked by western immunoblotting of undigested and digested extracts with an anti-Ub antibody. Quantification of ubiquitin content was performed by submitting Usp2-digested extracts to solid phase immunoassay as previously reported^28^. Briefly, protein samples were serially diluted with 140 mM NaCl so that the signal obtained after staining was linear with the amount applied and within the linear range of purified ubiquitin (from 10 to 0.6 ng) used as the reference standard. Samples and standard ubiquitin dilutions were loaded onto a nitrocellulose filter (0.2 μm pore size, BioRad) with the aid of a 96-well Dot Blot apparatus (BioRad) and processed as described^28^. Fixed blots were immunochemically stained for ubiquitin. Detection and densitometric quantification were performed in a Chemidoc apparatus (BioRad) equipped with the Quantity One software.

### Electrophoretic mobility shift assay (EMSA)

Nuclear extracts were prepared as described above. Synthetic ODNs (HPLC-purified) were purchased from Thermo Fisher Scientific GmbH (Ulm, Germany) and their sequences (both wild-type and mutated) are depicted in the Supplementary Table S1. Double stranded oligonucleotides were 5′ end-labeled with [γ-^32^P] ATP (Perkin Elmer Life Sciences, Boston, USA) and T4 polynucleotide kinase (T4 PNK, Roche Diagnostics, Mannheim, Germany). For direct binding experiments, nuclear extracts (5 μg) were preincubated with 3 μg of double-stranded non-specific DNA competitor poly(dI-dC) (Amersham Pharmacia Biotech) for 10 min on ice in binding buffer^42^. After this time, a ^32^P-end-labeled DNA probe was added to the mixtures at a final concentration of 4 nM and the incubation was continued for an additional 30 min. Reaction mixtures were then submitted to electrophoretic separation on 5% native polyacrylamide gels. DNA/protein complexes were detected by exposing the dried gel in a Molecular Imager (Bio-Rad). For competition experiments, nuclear extracts were incubated with a 50-fold excess of double stranded competitor ODN for 10 min before adding the ^32^P-labeled probe.

### Chromatin immunoprecipitation (ChIP)

ChIP assay was performed using the EZ-ChIP^TM^ Assay kit (Upstate Biotechnology Inc., New York, NY, USA), essentially according to the manufacturer’s instructions, as described^39^. Briefly, HeLa cells were transfected with the Ubwt expression vector or the empty vector pCMV-Myc (also shortened as Myc). Two days after, cells were cross-linked with 1% formaldehyde and cross-linked DNA underwent twelve 15 s sonication pulses at 45 watts by using a Labsonic 1510 Sonicator (Braun, Melsungen, Germany), to obtain sheared chromatin with an average size of 200/500 bp. For each immunoprecipitation, 2 × 10^6^ cell equivalents of sheared chromatin were incubated overnight at 4 °C with 10 μg of specific antibodies (listed below), or with no antibody (negative control). Immunoprecipitated chromatin (bound fractions) and an aliquot of input chromatin (1%) were subjected to de-crosslinking and DNA purification. ChIPed DNA was then analyzed using the SYBR green Real-Time master mix described above and the primer sets reported in the Supplementary Table S1. Cycling conditions were as described above for gene expression studies. Raw data (threshold cycle, Ct) of promoter-specific amplifications from ChIPed DNA (IP sample) were expressed as % of chromatin input controls, calculated using the formula 2^−ΔCt^ × 100, where ΔCt = Ct_IP sample_ - Ct_input_. ChIP antibodies used in this study were: anti-YY1 (sc-281X ), anti-HSF1 (sc-9144X) and anti-HSF2 (sc-13056X) from Santa Cruz Biotechnology; anti-H2AUb (Lys 119) (D27C4) and anti-H2BUb (Lys 120) (D11) from Cell Signaling Technology; anti-H3ac (pan-acetyl) (28612004) and anti-H3K4me3 (12613005) from Active motif.

### Metabolic labeling of nascent transcripts

To analyze gene expression changes among the pool of nascent *UBC* mRNAs, we adopted the Click-iT Nascent RNA Capture kit (Thermo Fisher Scientific) and performed experiments essentially according to manufacturers’ instructions, with slight modifications. HeLa cells transfected with the Ubwt expression construct or the empty vector pCMV-Myc were pulsed (48 h post-transfection) either with 0.5 mM 5-ethynyl Uridine for 0.5 hours or with 0.2 mM 5-ethynyl Uridine overnight (∼14 hours). Cells were then collected for RNA isolation, performed with the RNeasy Plus Mini kit (Qiagen) described above. Newly synthesized transcripts were then subjected to biotinylation, which creates a biotin-based handle for capturing nascent RNA transcripts on streptavidin magnetic beads. After precipitation and quantification of RNA yield by Nanodrop, the biotinylated RNA was selected through binding to streptavidin coated beads and finally cDNA synthesis was performed “on beads”. The Superscript III First-Strand cDNA synthesis system (Thermo Fisher Scientific) has been employed for reverse transcription of RNA, following the provided protocol, with the exception that the bead suspension containing the template RNA was heated 5 min at 68–70 °C before primers annealing and the reaction mixtures were gently mixed during the reverse transcriptase (RT) reaction (1 h at 50 °C) to prevent the beads from settling. The RT reaction was terminated and cDNA released from the beads by heating the mixture at 85 °C for 5 minutes. The beads were immobilized by using a DynaMag Spin magnet while collecting the supernatant containing cDNA. Undiluted cDNA was used in qPCR (2 μL cDNA were added in a 25 μL amplification reaction) executed with the SYBR Green Real-Time master mix reported above.

### Nuclei purification and detection of unspliced transcripts

Cell nuclei were harvested by gentle lysis of the plasma membrane and low-speed centrifugation as detailed in ref. ^72^. Briefly, HeLa cells transfected in six well-plates were washed with ice-cold PBS and then harvested by mechanical scraping by adding 0.5 mL ice-cold PBS/well. Scraped cells were transferred to a 1.5 mL microcentrifuge tube, placed on ice, mixed by pipetting to ensure a uniform cell suspension and counted using the Countess^™^ II FL Automated Cell Counter (ThermoFisher Scientific). At least 10^6^ cells were pelleted by centrifugation at 400 × *g* for 4 min at 4 °C and supernatant was then discarded. The pellet was resuspended in 0.5 mL NP-40 Lysis Buffer (10 mM Tris-HCl pH 7.4, 10 mM NaCl, 3 mM MgCl_2_, 0.5% NP-40) and incubated on ice for 5 min. Nuclei were pelleted by centrifugation at 300 × *g* for 4 min at 4 °C. Supernatant was saved to extract RNA from the cytosol fraction, while the nuclear pellet was resuspended in a further 0.5 mL of NP-40 Lysis Buffer and immediately pelleted by centrifugation at 300 × *g* for 4 min at 4 °C. After collection of the supernatant as above, the nuclear pellet was resuspended in 350 μL RLT lysis buffer for RNA extraction. In the same way, 2 volumes of RLT were added to the harvested cytosol fraction. RNA samples were purified from the nuclear and cytoplasmic fractions using the RNeasy Plus Mini kit and then analyzed by denaturing agarose gel electrophoresis. The images were captured using a gel documentation system (Bio-Rad, Hercules, CA, USA). Reverse transcription was performed with the PrimeScript^TM^ RT Master Mix. qPCR assays were carried out with the SYBR Green Real-Time master mix referred to above and new suitably designed primer sets for detecting nascent transcripts. These primers, named “NRO primers” (as in the reference paper of Roberts *et al*.^72^ from which some sequences have been picked and slightly modified) have been designed in order to fall within introns or to span an intron-exon boundary. The sequence as well as the position of these NRO primers are reported in the Supplementary Table S1.

### Statistics

Statistical analyses for experimental data were performed with PRISM software (GraphPad Software, La Jolla, CA, USA). Statistical significance was evaluated by the two-tailed paired Student’s t test for pairwise comparisons or one-way ANOVA with Tukey-Kramer multiple comparisons test for multiple comparisons. Results were expressed as means ± SEM and differences between values were considered significant for p < 0.05.

## Supplementary information

Supplementary Information

## Data Availability

All data generated or analysed during this study are included in this published article (and its Supplementary Information Files).
