# Supplementary material for: A negative feedback mechanism links UBC gene expression to ubiquitin levels by affecting RNA splicing rather than transcription
Source: Sci Rep. 2019 Dec 6;9:18556. doi: 10.1038/s41598-019-54973-7 (PMC6898720; doi:10.1038/s41598-019-54973-7)
Supplement: Supplementary file 1 — Supplementary Information [file 41598_2019_54973_MOESM1_ESM.pdf]

Supplementary Information for

A negative feedback mechanism links *UBC* gene expression to ubiquitin levels by affecting RNA splicing rather than transcription

Marzia Bianchi*, Rita Crinelli, Elisa Giacomini, Elisa Carloni, Lucia Radici, Emanuele-Salvatore Scarpa, Filippo Tasini and Mauro Magnani

Department of Biomolecular Sciences
University of Urbino *Carlo Bo*, 61029 - Urbino (PU), Italy

* To whom correspondence should be addressed
marzia.bianchi@uniurb.it
Tel.: +39 0722 305252
Fax: +39 0722 305324

SUPPLEMENTARY FIGURE LEGENDS

Supplementary Figure S1

HSF2 total protein levels in HeLa cells transfected with the indicated constructs.

Quantification of HSF2 protein factor in whole extracts obtained from HeLa cells transiently transfected with the indicated Ub expression vectors or the empty control vector (Myc). Relative amounts are expressed vs. the control Myc, set to 1. The histogram shows the means ± SEM of three independent experiments.

Supplementary Figure S2

Previous characterization of *UBC* promoter and reporter constructs used in this study.

(**A**) Schematic representation of the *UBC* promoter region previously investigated, with the *trans*-acting factors driving promoter activity under basal and stressful conditions. YY1 binding sites are present both in the intron and in the upstream promoter sequence[39]. Likewise, Sp1 binding sites have been identified both upstream of the TSS[43] and within the intron sequence[38,39]. HSF1 and HSF2 interact with HSEs in the upstream promoter region[16,42]. (**B**) The P916 wild type reporter construct has been used as the template to mutagenize the three HSEs in the upstream promoter, alone or in combination, to generate the constructs referred to as: P916 HSF mut a, b, a-b. The P371 wild type reporter construct has been used as the template to mutagenize the Sp1 and YY1 motifs identified in the intron, alone or in combination, to generate the following constructs: P371 Sp1 mut a, b, c, d, a-d; P371 YY1 mut a, b, a-b.

Supplementary Figure S3

Preparation and validation of nuclear extracts.

(**A**) Nuclei were harvested by gentle lysis and low speed centrifugation as described[72]. Whole cells and nuclear extracts from either Myc or Ub transfected samples were visualized by light microscopy (using a 40X objective). (**B**) Proteins harvested from nuclear fractions (7.5 and 15 μg in lanes 1 and 2, respectively) were subjected to immunoblotting analysis with anti-Lamin A/C and anti-GAPDH antibodies. The position of molecular mass markers is indicated on the left. (**C**) RNA was extracted from whole cells (total) and from both the nuclear and cytosolic fractions. The 28S and 18S ribosomal RNAs were visualized by running 10 μL of each elution (of 50 μL) on a 1.3% formaldehyde-agarose gel. The image shown in (A) is representative of three independent experiments. For (B) and (C), representative gels, relative to the Myc-transfected sample, are shown. Full-length immunoblots and gel image are presented in Supplementary Figure S9.

Supplementary Figure S4

Determination of *UBC* mRNA half-life.

(**A,B**) RTqPCR analysis of *UBC* mRNA in HeLa cells transfected with the Ubwt expression construct or the empty vector Myc, at indicated times after treatment with Actinomycin D (ActD). The half-life was calculated by the equation $T_{1/2} = \ln 2/K_{decay}$. Data presented in the graphs are means ± SEM of three independent experiments; $p = 0.5933$ vs. Myc.

Supplementary Figure S5

Full immunoblot exposures for results shown in Figure 2A-2B-2C.

Lanes highlighted by a star represent samples unrelated to this study.

Supplementary Figure S6

Full-length immunoblots for results shown in Figure 4A.

Lanes highlighted by a star represent samples unrelated to this study.

Supplementary Figure S7
Full-length immunoblots and EMSA for results shown in Figure 5A and 5B.
Lanes highlighted by a star represent samples unrelated to this study.

Supplementary Figure S8
Full-length immunoblots and EMSA for results shown in Figure 6A and 6B.
Lanes highlighted by a star represent samples unrelated to this study.

Supplementary Figure S9
Full-length immunoblots and gel image for results shown in Supplementary Figure S3B and S3C.

REFERENCES CITED IN THE SUPPLEMENTARY FILE (numbers refer to the REFERENCE list of the Manuscript)

16. Bianchi, M., Crinelli, R., Arbore, V. & Magnani, M. Induction of ubiquitin C (UBC) gene transcription is mediated by HSF1: role of proteotoxic and oxidative stress. *FEBS Open Bio* **8,** 1471-1485, https://doi.org/10.1002/2211-5463.12484 (2018).
38. Bianchi, M., Crinelli, R., Giacomini, E., Carloni, E. & Magnani, M. A potent enhancer element in the 5'-UTR intron is crucial for transcriptional regulation of the human ubiquitin C gene. *Gene* **448,** 88-101, https://doi.org/10.1016/j.gene.2009.08.013 (2009).
39. Bianchi, M. *et al*. Yin Yang 1 intronic binding sequences and splicing elicit intron-mediated enhancement of ubiquitin C gene expression. *PLoS One* **8,** e65932, https://doi.org/10.1371/journal.pone.0065932 (2013).
42. Crinelli, R. *et al*. Molecular Dissection of the Human Ubiquitin C Promoter Reveals Heat Shock Element Architectures with Activating and Repressive Functions. *PLoS One* **10,** e0136882, https://doi.org/10.1371/journal.pone.0136882 (2015).
43. Marinovic, A.C., Zheng, B., Mitch, W.E. & Price, S.R. Ubiquitin (UbC) expression in muscle cells is increased by glucocorticoids through a mechanism involving Sp1 and MEK1. *J. Biol. Chem*. **277,** 16673-16681 (2002).
72. Roberts, T.C. *et al*. Quantification of nascent transcription by bromouridine immunocapture nuclear run-on RT-qPCR. *Nat. Protoc*. **10,** 1198-1211, https://doi.org/10.1038/nprot.2015.076 (2015).

Supplementary Figure S1

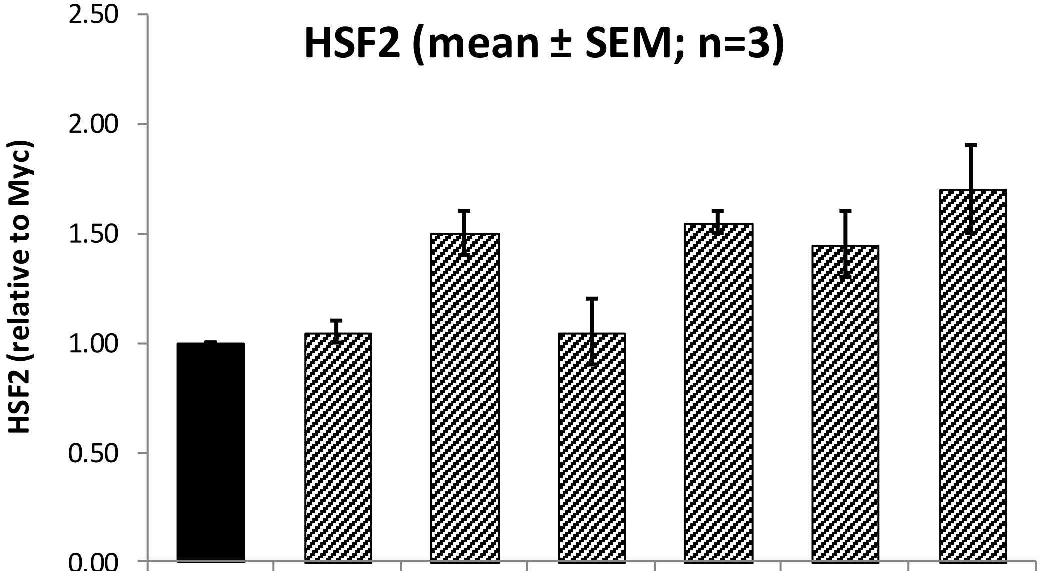

	Myc	Ubwt	UbK48R	UbK63R	UbG76A	UbI44A	UbDGG
HSF2	1.00	1.05	1.50	1.05	1.55	1.45	1.70

Supplementary Figure S2

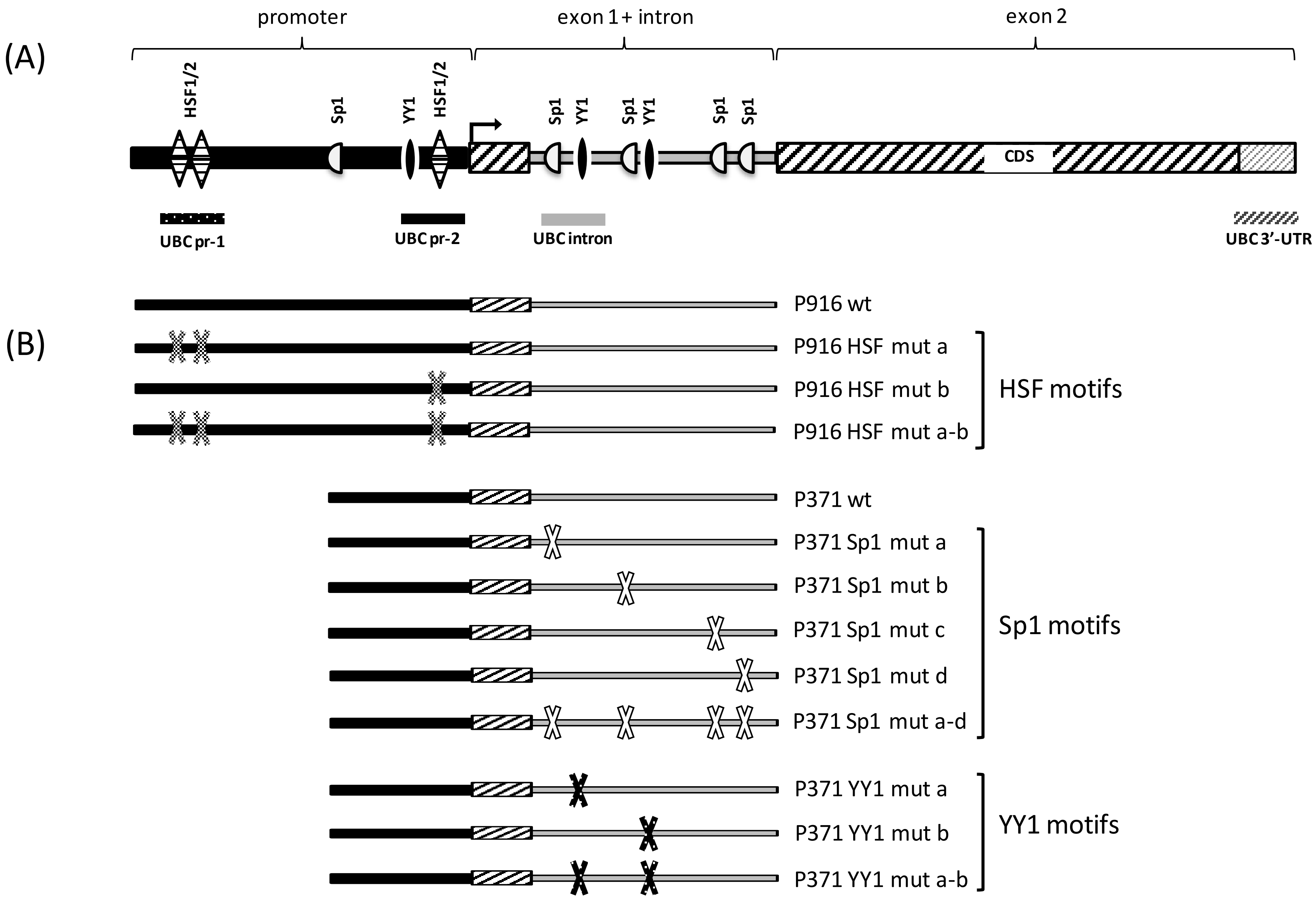

Supplementary Figure S3

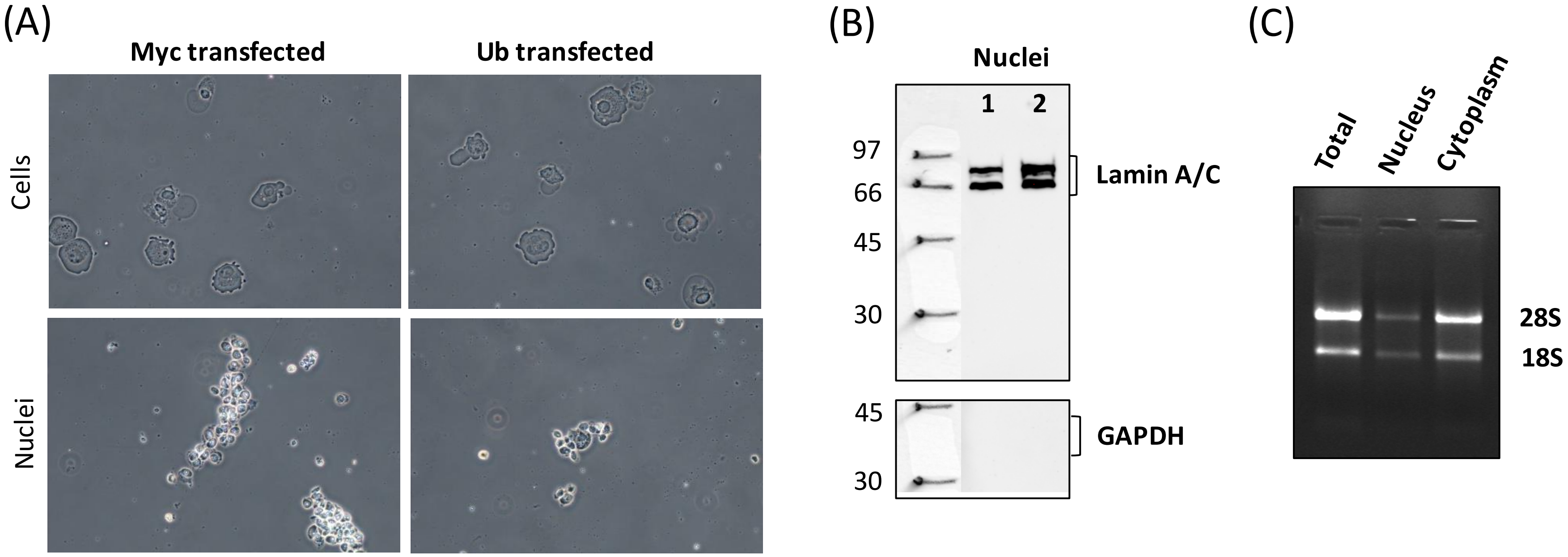

Supplementary Figure S4

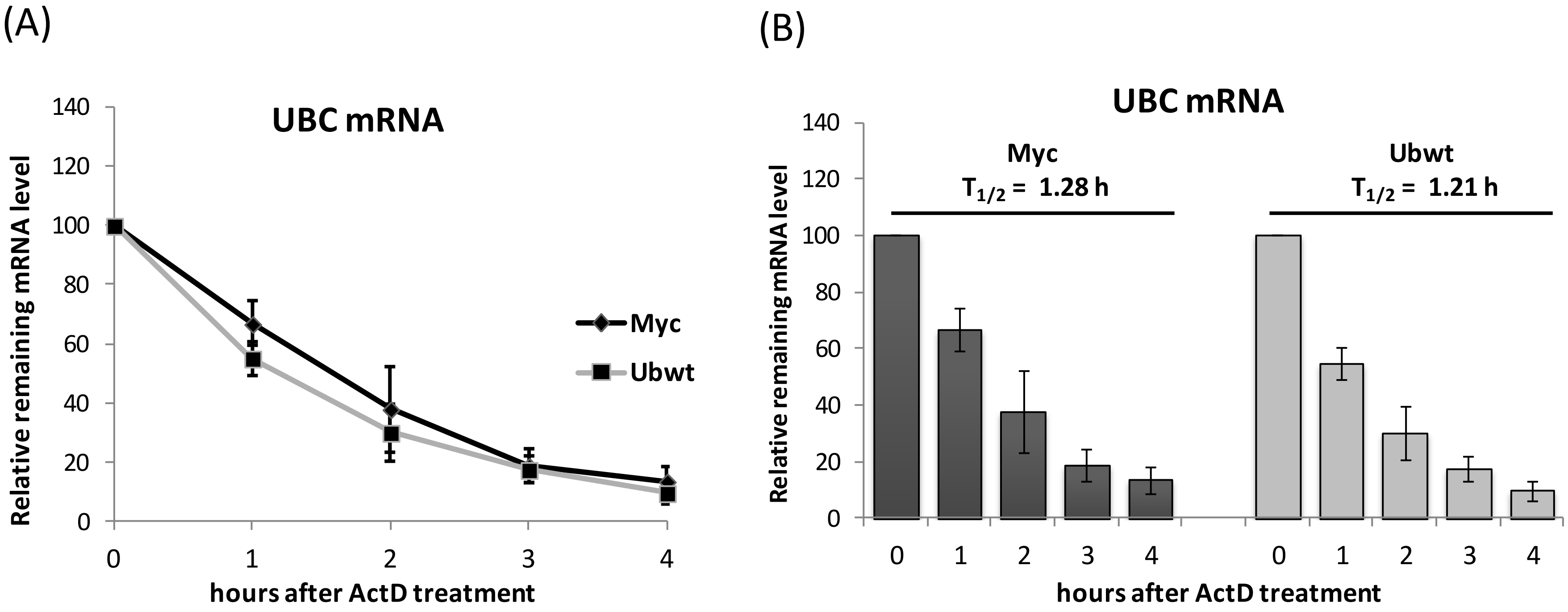

half life ($T_{1/2}$) = ln2 /K_{decay}

Supplementary Figure S5

Fig. 2A

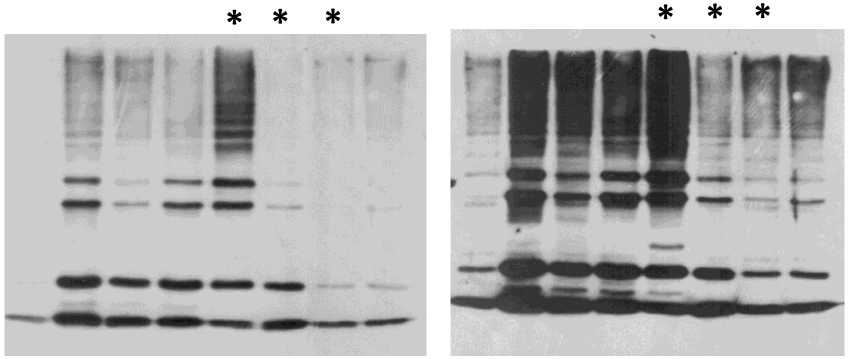

Low exposure High exposure

Fig. 2B

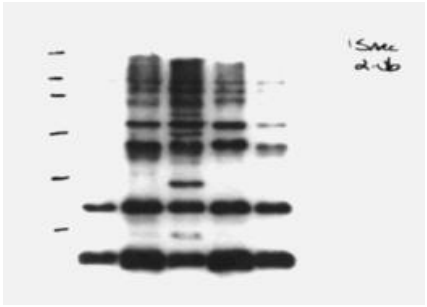

Fig. 2C

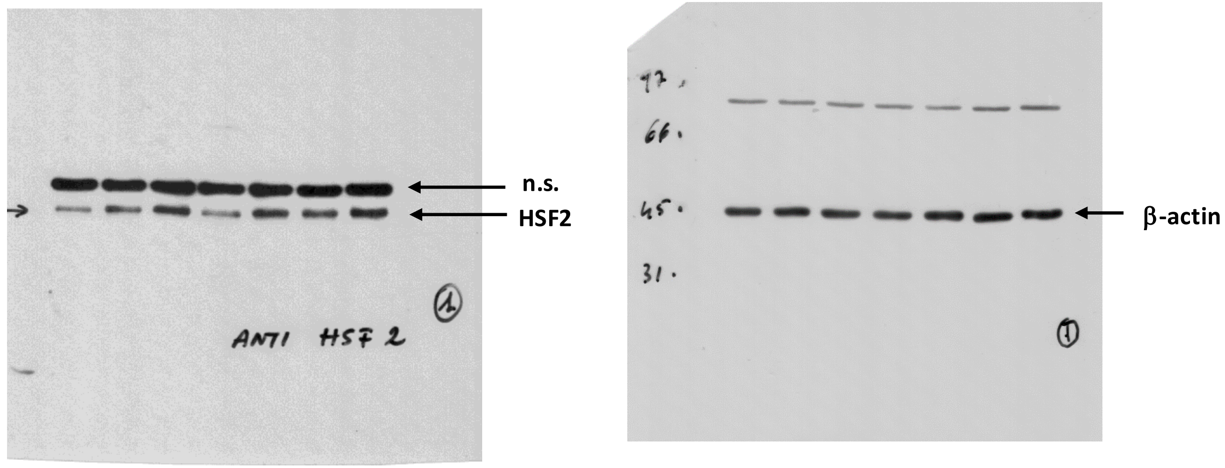

Supplementary Figure S6

Fig. 4A

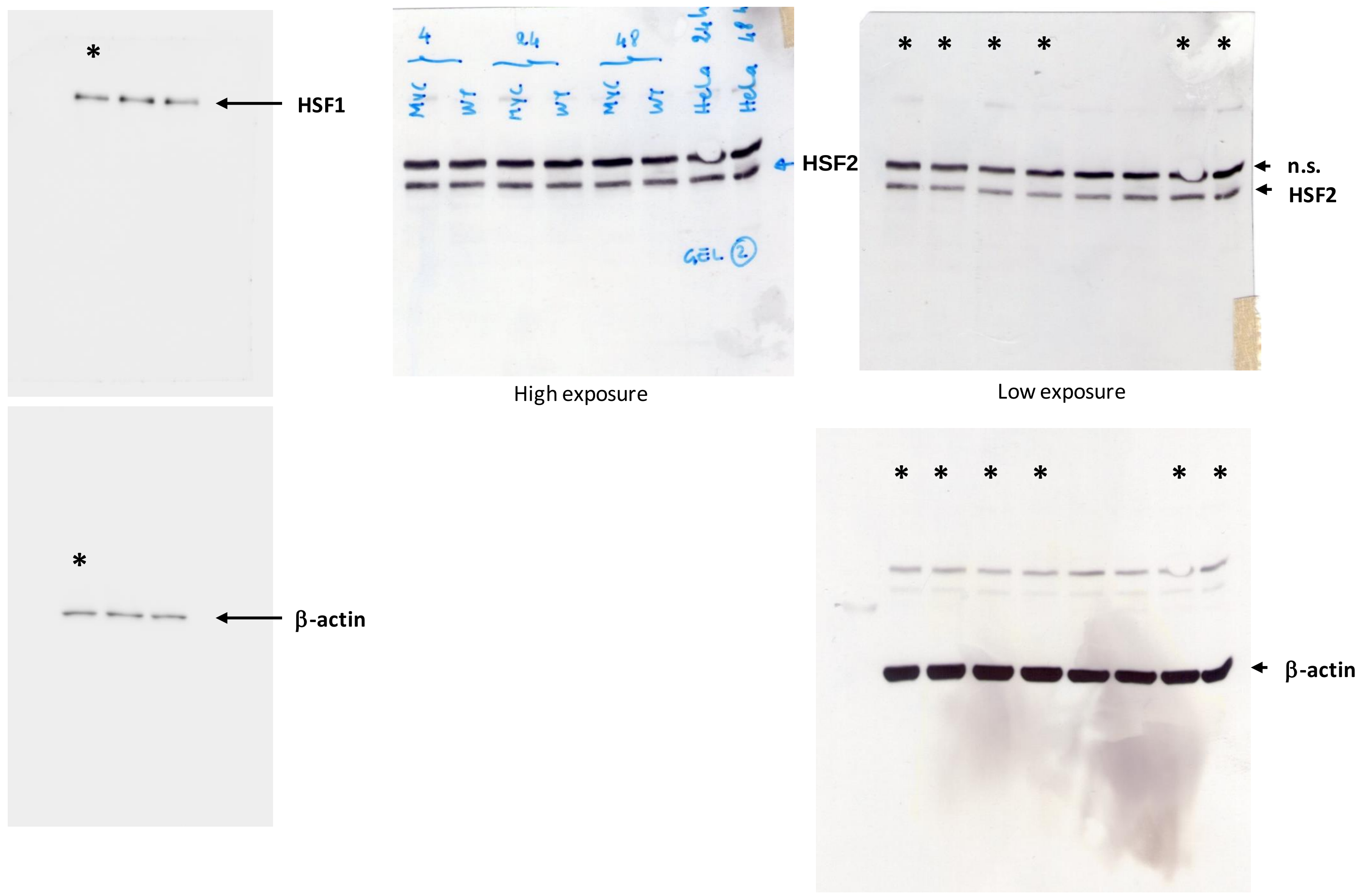

Supplementary Figure S7

Fig. 5A

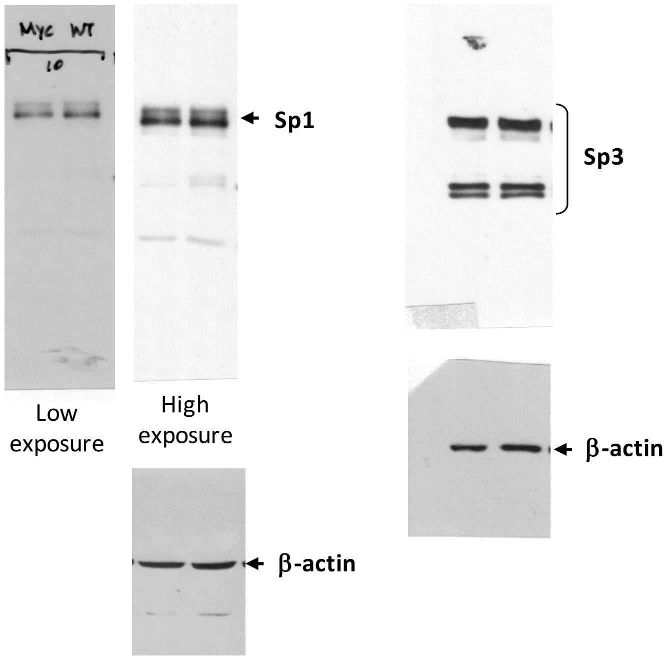

Fig. 5B

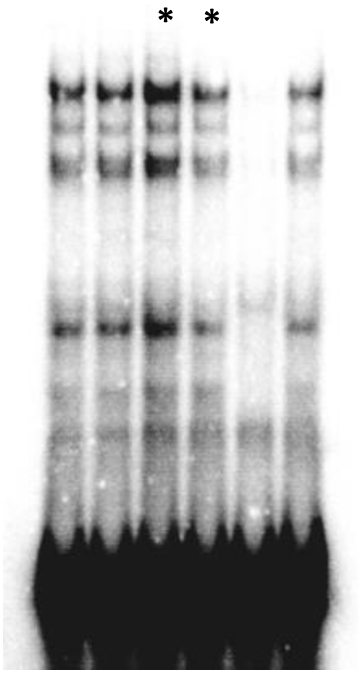

Supplementary Figure S8

Fig. 6A

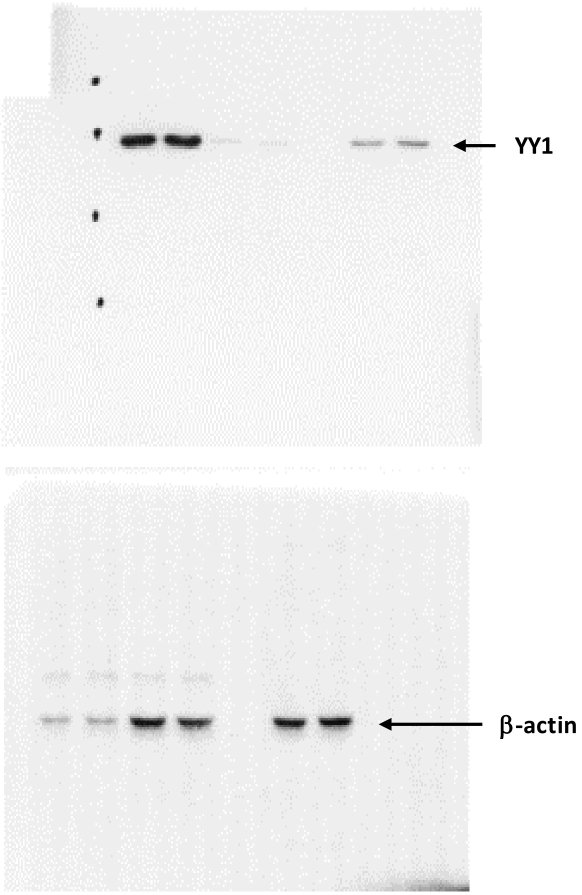

Fig. 6B

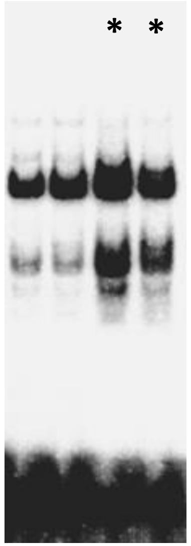

Supplementary Figure S9

Fig. S3B

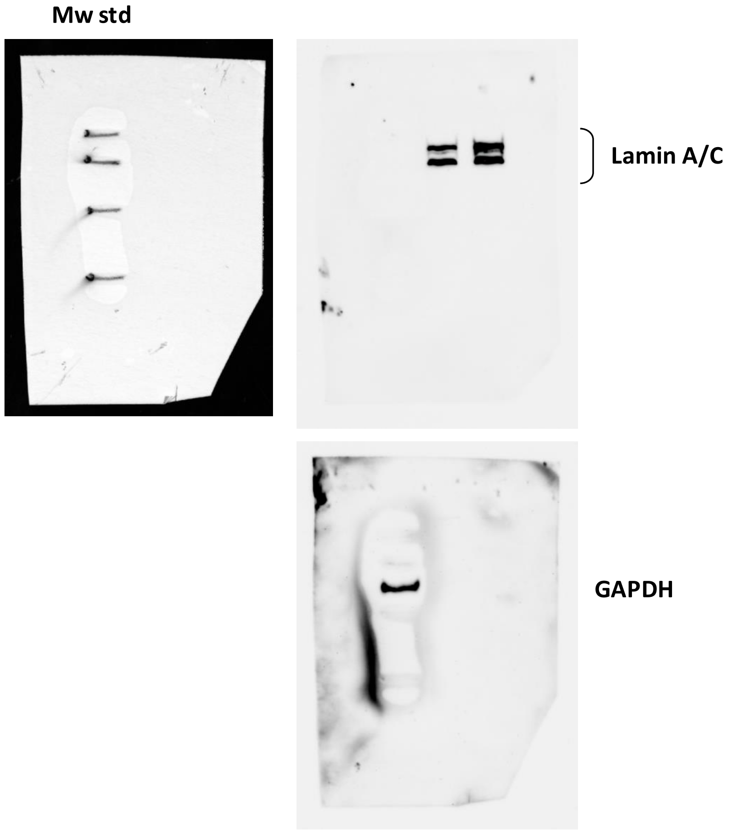

Fig. S3C

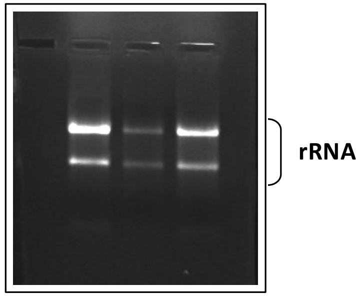

Supplementary Table S1. Synthetic oligonucleotides used in this study

oligo ID_strand	SEQUENCE (5’ to 3’)	APPLICATION	NOTES	REFERENCE
Oligonucleotides used for reporter and ubiquitin expression constructs			*Restriction enzyme cutting site*	
hUBC (-916)_F	ggtaccGAGCTCGAGAAATTTCCATGCCTCCCTG	*cloning*	*Sac I*	*Bianchi et al., Gene 2009*
hUBC (-535)_F	caggtaccGAGCTCAGACCCGTCCATCTCGCAGG	*cloning*	*Sac I*	*Bianchi et al., Gene 2009*
hUBC (-371)_F	caggtaccGAGCTCTAAGGAACGCGGGCCGCCCA	*cloning*	*Sac I*	*Bianchi et al., Gene 2009*
hUBC (-254)_F	ggtaccGAGCTCAGCGAGCGTCCTGATCCTTC	*cloning*	*Sac I*	*This study*
hUBC (-195)_F	ggtaccGAGCTCCTCGGCCTTAGAACCCCAGTATC	*cloning*	*Sac I*	*This study*
hUBC (-123)_F	ggtaccGAGCTCTTTCTTTCCAGAGAGCGGAACAG	*cloning*	*Sac I*	*This study*
hUBC (-84)_F	caggtaccGAGCTCCCTTCTCGGCGATTCTGCGG	*cloning*	*Sac I*	*This study*
hUBC (-37)_F	ggtaccGAGCTCGATGATTATATAAGGACGCG	*cloning*	*Sac I*	*Bianchi et al., PLoS One 2013*
hUBC (+4)_R	gtccatggAAGCTTAACTAGCTGTGCCACACCCG	*cloning*	*Hind III*	*Bianchi et al., Gene 2009*
hUBC (+63)_R	agatctGCTAGCAAGTGACGATCACAGCGATCCAC	*cloning*	*Nhe I*	*Bianchi et al., Gene 2009*
hUBC (+876)_R	tggAAGCTTGTCTAACAAAAAAGCCAAAAACGGC	*cloning*	*Hind III*	*Bianchi et al., Gene 2009*
UbΔGG_F	GTACCCGCGGGCCCATGCAGATCTTCGTGAAG	*cloning*	*Apa I*	*This study*
UbΔGG_R	TTCGGCTTGGTACC**TCA**TCTAAGACGGAGCACCAG	*cloning*	*Kpn I*	*This study*
UbK0_F	GTGGTGGGGGGCCCATGCAGATCTTCGTCAG	*cloning*	*Apa I*	*This study*
UbK0_R	TTAGCGGCGGTACCACCACCTCTTAGTCTTAAGACAAG	*cloning*	*Kpn I*	*This study*
UbI44A_F	CCTGACCAGCAGAGGTTG**GC**CTTTGCTGGGAAACAGCT	*mutagenesis*		*This study*
UbI44A_R	AGCTGTTTCCCAGCAAAG**GC**CAACCTCTGCTGGTCAGG	*mutagenesis*		*This study*
Kozak_UbK0_F	TGCGGAATTGTACCCGCC**CCACC**ATGCAGATCTTCGTCAG	*mutagenesis*		*This study*

Oligonucleotides used for gene expression studies by RTqPCR				
UBC_F	GTGTCTAAGTTTCCCCTTTTAAGG	*real time*		*Bianchi et al., PLoS One 2013*
UBC_R	TTGGGAATGCAACAACTTTATTG	*real time*		*Bianchi et al., PLoS One 2013*
UBB_F	CTTTGTTGGGTGAGCTTGTTTGT	*real time*		*Bianchi et al., Gene 2015*
UBB_R	GACCTGTTAGCGGATACCAGGAT	*real time*		*Bianchi et al., Gene 2015*
UBA52_F	CTGCGAGGTGGCATTATTGAG	*real time*		*Bianchi et al., Gene 2015*
UBA52_R	GTTGACAGCACGAGGGTGAAG	*real time*		*Bianchi et al., Gene 2015*
RPS27A_F	TCGTGGTGGTGCTAAGAAAAGG	*real time*		*Bianchi et al., Gene 2015*
RPS27A_R	TTCAGGACAGCCAGCTTAACCT	*real time*		*Bianchi et al., Gene 2015*
HSP70_F	AGCTGAAGAAGGGTCAAGTGAC	*real time*		*Bianchi et al., FEBS Open Bio 2018*
HSP70_R	TGGATAGGGCAAATCCTGAG	*real time*		*Bianchi et al., FEBS Open Bio 2018*
GAPDH_F	TGCACCACCAACTGCTTAG	*real time*		*Crinelli et al., PLoS One 2015*
GAPDH_R	GATGCAGGGATGATGTTC	*real time*		*Crinelli et al., PLoS One 2015*
B2M_F	GCCTGCCGTGTGAACCAT	*real time*		*Bianchi et al., PLoS One 2013*
B2M_R	CATCTTCAAACCTCCATGATGCT	*real time*		*Bianchi et al., PLoS One 2013*
LUC_F	TGTACACGTTCGTCACATCTCATCT	*real time*		*Bianchi et al., PLoS One 2013*
LUC_R	AGTGCAATTGTCTTGTCCCTATCG	*real time*		*Bianchi et al., PLoS One 2013*
Oligonucleotides used for detection of unspliced RNA			*Primer position*	
nro-UBC_F	GGATTTGGGTCGCAGTTCTT	*real time unspliced*	exon 1	*This study*
nro-UBC_R	TTGGCGGTCTCTCCACAC	*real time unspliced*	intron	*This study*
nro-UBB_F	GGCATTTTGAAGGAATAGTTGC	*real time unspliced*	intron	*This study*
nro-UBB_R	ACGAAGATCTGCATTTTGACCT	*real time unspliced*	intron-exon junction	*This study*

nro-GAPDH_F	AATCCCATCACCATCTTCCAG	*real time unspliced*	exon	*This study*
nro-GAPDH_R	GGAGCCACACCATCCTAGTTG	*real time unspliced*	intron	*This study*
nro-B2M_F	GACACCAAGTTAGCCCCAAG	*real time unspliced*	intron	*This study*
nro-B2M_R	AACCCAGACACATAGCAATTCAG	*real time unspliced*	exon	*This study*
Oligonucleotides used for ChIP			*Previous name*	
UBC pr1_F	GAGAAATTTCCATGCCTCCCTGTT	*real time*	FR1 (-916) fwd	*Crinelli et al., PLoS One 2015*
UBC pr1_R	AAAAGAGGCGGAAACCCCACA	*real time*	FR1 (-759) rev	*Crinelli et al., PLoS One 2015*
UBC pr2_F	ACTCGGCCTTAGAACCCCAGTA	*real time*	FR6 ChIP forward	*Crinelli et al., PLoS One 2015*
UBC pr2_R	CTCGCCTGTTCCGCTCTCT	*real time*	FR6 ChIP reverse	*Crinelli et al., PLoS One 2015*
UBC intron_F	ACCGCCAAGGGCTGTAGTCT	*real time*		*This study*
UBC intron_R	CTCACAAGCGTCTTCCATTCAAG	*real time*		*This study*
UBC 3'-UTR_F	GTGTCTAAGTTTCCCCTTTTAAGG	*real time*	The same used for gene expression studies (UBC_F)	*Bianchi et al., PLoS One 2013*
UBC 3'-UTR_R	TTGGGAATGCAACAACTTTATTG	*real time*	The same used for gene expression studies (UBC_R)	*Bianchi et al., PLoS One 2013*
Oligonucleotides used for EMSA			*Primer position*	
Sp1_wt	TTTTGGCGCCTCCCGCGGGCGCCCCCCTCCTCACGGCGAG	*EMSA*	human *UbC* (−319 to −280)	*Marinovic et al., J. Biol. Chem. 2002*
Sp1_mut	TTTTGG**A**G**AA**TCCCGCGGGCGCC**TA**C**G**TCCTCACGGCGAG	*EMSA*	human *UbC*mut2x (−319 to −280)	*Marinovic et al., J. Biol. Chem. 2002*
YY1 intron probe	AGCAAAATGGCGGCTGTTCCCGAGTCT	*EMSA*	intron	*This study*

F, forward; R, reverse.
Regarding the primers used for reporter constructs: the numbers in brackets refer to the position respect to the transcription start site (TSS) of *UBC* gene identified as +1; the lower case letters stand for degenerate extra-sequences; the restriction enzyme sites are underlined.
For the nro- (Nuclear Run On) primers, the position of the binding sequence is indicated in the NOTES column. The letters in bold in the mutagenesis primers as well as in the Sp1_mut oligonucleotide used in EMSA indicate the nucleotide changes introduced.

Supplementary Table S2. Plasmids used in this study

CONSTRUCT ID_ (CURRENT NAME)	DESCRIPTION	PREVIOUS NAME	REFERENCES
Expression constructs			
Ubwt	expression of wild-type ubiquitin (Ub)	Ub-Myc^{cl}	*Crinelli et al., Mol. Cell. Biochem. 2008*
UbK48R	expression of Ub carrying the Lys^{48}→Arg^{48} substitution	UbK48R	*Crinelli et al., Mol. Cell. Biochem. 2008*
UbK63R	expression of Ub carrying the Lys^{63}→Arg^{63} substitution	UbK63R	*Crinelli et al., Mol. Cell. Biochem. 2008*
UbK0	expression of Ub carrying all the Lys replaced with Arg (Lys-less Ub)		*Lim et al., J. Neurosci. 2005*
UbG76A	expression of Ub carrying the Gly^{76}→Ala^{76} substitution	UbG76A	*Palma et al., Mol. Cell. Biochem. 2009*
UbΔGG	expression of Ub lacking the Gly^{75} and Gly^{76} residues		*This study*
UbI44A	expression of Ub carrying the Ile^{44}→Ala^{44} substitution		*This study*
pCMV-Myc	empty vector	pCMV-Myc	*Crinelli et al., Mol. Cell. Biochem. 2008*
Reporter constructs			
5' and 3' serial deletions	*The cloned UBC promoter region is indicated in square brackets*		
P916	[-916/+876] intron included	P1	*Bianchi et al., Gene 2009*
P535	[-535/+876] intron included	P2	*Bianchi et al., Gene 2009*
P371	[-371/+876] intron included	P3	*Bianchi et al., Gene 2009*
P254	[-254/+876] intron included		*This study*
P195	[-195/+876] intron included		*This study*
P123	[-123/+876] intron included		*This study*
P84	[-94/+876] intron included		*This study*
P37	[-37/+876] intron included	P3 Δ[-371/-38] nt construct	*Bianchi et al., PLoS One 2013*

P916-int	[-916/+4] intron excluded	P4	*Bianchi et al., Gene 2009*
P535-int	[-535/+4] intron excluded	P5	*Bianchi et al., Gene 2009*
P371-int	[-371/+4] intron excluded	P6	*Bianchi et al., Gene 2009*
P371+chimeric int	[-371/+4] + chimeric intron	P7+chimeric intron	*Bianchi et al., Gene 2009*
pGL3-Basic	empty (promoter-less) vector	pGL3-Basic	*Bianchi et al., Gene 2009*
Mutagenized constructs			
P916 wt	wild type sequence	P1 (FR1-2-6)	*Crinelli et al., PLoS One 2015*
P916 HSF mut a	upstream HSF1/2 sites mutagenized	P1mut FR1-2	*Crinelli et al., PLoS One 2015*
P916 HSF mut b	proximal HSF1/2 site mutagenized	P1mut FR6	*Crinelli et al., PLoS One 2015*
P916 HSF mut a-b	both upstream and proximal HSF1/2 sites mutagenized		*This paper*
P371 wt	wild type sequence	P3	*Bianchi et al., Gene 2009*
P371 Sp1 mut a	first intronic Sp1 site mutagenized	Sp1 mut a	*Bianchi et al., PLoS One 2013*
P371 Sp1 mut b	second intronic Sp1 site mutagenized	Sp1 mut b	*Bianchi et al., PLoS One 2013*
P371 Sp1 mut c	third intronic Sp1 site mutagenized	Sp1 mut c	*Bianchi et al., PLoS One 2013*
P371 Sp1 mut d	fourth intronic Sp1 site mutagenized	Sp1 mut d	*Bianchi et al., PLoS One 2013*
P371 Sp1 mut a-d	all intronic Sp1 sites mutagenized	Sp1 mut a-d	*Bianchi et al., PLoS One 2013*
P371 YY1 mut a	first intronic YY1 site mutagenized	YY1 mut e	*Bianchi et al., PLoS One 2013*
P371 YY1 mut b	second intronic YY1 site mutagenized	YY1 mut f	*Bianchi et al., PLoS One 2013*
P371 YY1 mut a-b	all intronic YY1 sites mutagenized	YY1 mut e-f	*Bianchi et al., PLoS One 2013*

To make this part more understandable for readers, we have provided for each plasmid construct both the name by which it is referred to in this paper and the name used in previous publications (see REFERENCE column).
